# Targeting metabolic reprogramming to overcome drug resistance in advanced bladder cancer: insights from gemcitabine‐ and cisplatin‐resistant models

**DOI:** 10.1002/1878-0261.13684

**Published:** 2024-06-14

**Authors:** Ichiro Kawahara, Hirofumi Yoshino, Wataru Fukumoto, Junya Arima, Saeki Saito, Gang Li, Ikumi Fukuda, Akihiko Mitsuke, Takashi Sakaguchi, Satoru Inoguchi, Ryosuke Matsushita, Masayuki Nakagawa, Shuichi Tatarano, Yasutoshi Yamada, Hideki Enokida

**Affiliations:** ^1^ Department of Urology, Graduate School of Medical and Dental Sciences Kagoshima University Japan

**Keywords:** erdefitinib, FASN, FGFR, HIF1α, metabolism, PHGDH

## Abstract

Gemcitabine plus cisplatin (GC) combination chemotherapy is the primary treatment for advanced bladder cancer (BC) with unresectable or metastatic disease. However, most cases develop resistance to this therapy. We investigated whether drug resistance could be targeted through metabolic reprogramming therapies. Metabolomics analyses in our lab's gemcitabine‐ and cisplatin‐resistant cell lines revealed increased phosphoglycerate dehydrogenase (*PHGDH*) expression in gemcitabine‐resistant cells compared with parental cells. Isocitrate dehydrogenase 2 (IDH2) gain of function stabilized hypoxia‐inducible factor1α (*HIF1α*) expression, stimulating aerobic glycolysis. In gemcitabine‐resistant cells, elevated fumaric acid suppressed prolyl hydroxylase domain‐containing protein 2/Egl nine homolog 1 (*PHD2*) and stabilized *HIF1α* expression. *PHGDH* downregulation or inhibition in gemcitabine‐resistant BC cells inhibited their proliferation, migration, and invasion. Cisplatin‐resistant cells showed elevated fatty acid metabolism, upregulating fatty acid synthase (*FASN*) downstream of tyrosine kinase. Using the fibroblast growth factor receptor (*FGFR*) tyrosine kinase inhibitor erdafitinib, we inhibited malonyl‐CoA production, which is crucial for fatty acid synthesis, and thereby suppressed upregulated HIF1α expression. Combination treatment with NCT503 and erdafitinib synergistically suppressed tumor cell proliferation and induced apoptosis *in vitro* and *in vivo*. Understanding these mechanisms could enable innovative BC therapeutic strategies to be developed.

## Graphical abstract

This study reveals a complex network of metabolic reprogramming in gemcitabine‐ and cisplatin‐resistant (GR and CR) bladder cancer (BC) cells. By targeting key players in the metabolic network, including *PHGDH*, *FASN*, and *HIF1α*, the authors pave the way for a deeper understanding of drug‐resistant BC. Combination treatment with NCT503 and erdafitinib synergistically suppressed tumor cell proliferation and induced apoptosis.

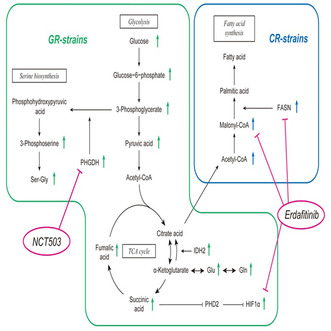

**Abbreviations**
BC: bladder cancer
CDDP: cisplatin
FASN: fatty acid synthase
FGFR: fibroblast growth factor receptor
GEM: gemcitabine
HIF1α: hypoxia‐inducible factor1α
IC50: half maximal (50%) inhibitory concentration
IDH2: isocitrate dehydrogenase 2
PHD2: prolyl hydroxylase domain‐containing protein 2
PHGDH: phosphoglycerate dehydrogenase
PRPP: phosphoribosyl pyrophosphate
TCGA: The Cancer Genome Atlas

## 1 Introduction

Bladder cancer (BC) is the 10th most diagnosed cancer worldwide, with 573 000 new cases and 213 000 deaths reported per year. It was also the third most common cancer in men and the sixth most common cause of cancer‐related death in 2020 [1]. BC is classified into non‐muscle and muscle layer invasive BC according to the degree of cancer invasion. Non‐muscle invasive BC accounts for 70–80% of cases and is almost always curable, with a 5‐year survival rate of approximately 90% [2, 3]. On the other hand, 20–30% of cases are muscle layer invasive BC, with a 5‐year survival rate of approximately 60–70% [3]; of these cases, approximately 10% are metastatic cancer, with a low 5‐year survival rate of approximately 6–30% [4]. Neoadjuvant and adjuvant chemotherapy consisting of gemcitabine and cisplatin is used as the primary treatment for advanced BC [5]. Gemcitabine plus cisplatin combination therapy has a complete response rate of 14.5% and partial response rate of 34.5%, but the median overall survival (9.8 months) after chemotherapy is poor [6]. Newer immune checkpoint inhibitors, including pembrolizumab, as first‐ or second‐line therapy for metastatic BC are clinically effective in some patients (response rate: 13–21% [7, 8, 9, 10]; overall survival: 10.3 months [8]). In addition, erdafitinib, an inhibitor of *FGFR*, showed efficacy as a second‐line treatment for drug‐resistant BC (response rate: 40%; [11, 12]; overall survival: 13.8 months [12]). Erdafitinib treatment also significantly prolonged overall survival over chemotherapy in patients with metastatic urothelial carcinoma and *FGFR* alterations who had previously been treated with immune checkpoint inhibitors [13]. Erdafitinib is particularly useful for patients with the luminal I subtype harboring *FGFR* mutations, which usually has low PD‐L1 expression and thus may not respond to immunotherapy [14].

On the other hand, resistance to chemotherapy or molecular targeted therapy is a major cause of tumor recurrence and death, and it is important to understand the metabolic reprogramming associated with drug resistance [15]. Metabolic changes are important features of cancer, and many factors mediate metabolic reprogramming, including oncogenes, growth factors, hypoxia‐inducible factors, and tumor suppressor genes. These changes cause alterations in cellular metabolism, particularly glucose metabolism, as glucose absorption is dramatically increased in cancer [16]. Metabolic reprogramming allows cancer cells to adapt to dramatic changes in the tumor environment. Tumors adapt to conventional antineoplastic therapy via chemotherapy resistance, residual disease, and tumor recurrence [17].

We previously reported that miR‐99a‐5p induces cellular senescence in gemcitabine‐resistant BC cells by targeting *SMARCD1* [18], and that miRNA‐486‐5p overcomes the resistance of cisplatin‐resistant BC cells by targeting *EHHADH* [19]. We found that gemcitabine‐ and cisplatin‐resistant BC cells did not show cross resistance, supported by different mRNA expression patterns in gemcitabine‐ versus cisplatin‐resistant cells [20]. On the other hand, we previously demonstrated that phosphoglycerate dehydrogenase (*PHGDH*), a regulator of the serine/glycine biosynthesis pathway, was upregulated in hypoxia‐inducible factor (*HIF*)2‐knockout sunitinib‐resistant renal carcinoma cells. This finding that metabolic reprogramming occurs in drug‐resistant cells suggests a potential therapeutic target [21]. However, metabolism‐based drug resistance mechanisms in BC have not been well studied, and our knowledge is still limited.

In this study, metabolomics analysis was performed in drug‐resistant BC cells, focusing on metabolites. Based on the results of the metabolomics analysis, we targeted key metabolic genes and metabolites and performed *in vitro* functional analyses. The *in vitro* results were then validated *in vivo*.

## 2 Materials and methods

### 2.1 Establishment of resistant cell lines *in vitro*

We used the two human BC cell lines T24 (RRID: CVCL_0554) and J82 (RRID: CVCL_0359), purchased from the American Type Culture Collection (ATCC, Manassas, VA, USA). These cell lines were validated by short tandem repeat (STR) testing by Promega Company (Tokyo, Japan). Mycoplasma was also negative. These cell lines were cultured in minimum essential medium (MEM) containing 10% FBS, 50 U·mL^−1^ penicillin, and 50 μg·mL^−1^ streptomycin at 37 °C in a humidified environment consisting of 95% air/5% CO_2_. To establish gemcitabine‐resistant T24 (GEM‐R‐T24) cells [18], previously established cells were cultured with 1–450 ng·mL^−1^ gemcitabine for 12 months, followed by 600 ng·mL^−1^ gemcitabine for 6 months in our laboratory; after 48 h of continuous culture, the surviving cells were collected and passaged to establish a resistant line. To establish cisplatin‐resistant T24 (CDDP‐R‐T24) cells [19], cells were cultured with 0.01–2 μg·mL^−1^ cisplatin for 6 months, followed by 3 μg·mL^−1^ cisplatin for another 6 months in our laboratory; after 48 h of continuous culture, the surviving cells were collected and passaged to establish a resistant line. Gemcitabine and cisplatin were continuously added to each resistant cell culture.

### 2.2 Establishment of resistant mouse strains *in vivo* and of a tumor xenograft model

The animal experiments described here were conducted in accordance with the Kagoshima University Regulations on Animal Experiments. Five‐week‐old female nude mice (BALB/c nu/nu) were purchased from Charles River Laboratories (Kumamoto, Japan). Each cell lines were used in three to four mice, and experiments were performed in accordance with the guidelines for animal welfare and use in cancer research. Mice were kept in a rectangular cage (225 × 338 × 140 mm) under standard experimental conditions (12‐h day/night cycle, 25 °C). The cages were cleaned once a week and were covered in sawdust to ensure water absorption and flexibility. The mice were provided unlimited water and a standard diet (CLEA Rodent Diet CL‐2, Tokyo Japan). BC cell lines (4 × 10^7^ mL^−1^; in 100 μL) were mixed with 100 μL Matrigel (BD Biosciences, Bedford, MA, USA). Two hundred microliters of parental BC cells (T24, J82) were injected subcutaneously into the lateral aspect (*n* = 3).

To acquire drug resistance, J82 BC cells were subcutaneously administered together with 150 mg·kg^−1^ gemcitabine (once a week) and 4 mg·kg^−1^ cisplatin (five times a week) into the mice, after which the mice were sacrificed and the tumors removed. Tumor size was calculated as major axis × minor axis 2 × (π/6). The excised tumors were washed with PBS, soaked in trypsin, chopped into small pieces using a scalpel, and exposed to 37 °C for 1 h. The tumor was then centrifuged to remove the trypsin, washed again in PBS, and centrifuged again, after which the tumor cells and medium were cultured and passaged.

To evaluate the efficacy of erdafitinib and NCT503, 4 × 10^6^ cisplatin‐resistant T24 BC cells were injected subcutaneously into the lateral aspect of female nude mice (BALB/c nu/nu, 6–8 weeks old). Viable mice were divided into three groups (4 per group). The following treatment regimens were used: vehicle, oral erdafitinib, and oral erdafitinib + intraperitoneal NCT503. Both 40 mg·kg^−1^ erdafitinib 5 days a week and 40 mg·kg^−1^ NCT503 5 days a week were administered starting on day 7 post‐xenograft. In the comparison between the vehicle group and the NCT503 alone group, the mice were divided into two groups (5 per group) and administered five times a week. Weight and tumor measurements were performed twice a week, and sacrifices were made on day 25 or 29. NCT503 (AOBIOUS, Gloucester, MA, USA) was used as a *PHGDH* inhibitor and erdafitinib (JNJ‐42756493; Selleck, Yokohama City, Japan) as an FGFR inhibitor.

### 2.3 Determination of the half maximal (50%) inhibitory concentration (IC50)

To determine the IC50, cells were seeded in triplicate in 96‐well plates at 2000/well, and gemcitabine‐/cisplatin‐resistant cells were treated with one treated with serially diluted concentration of gemcitabine and cisplatin. Similarly, the parental cells and the gemcitabine‐resistant cells were treated with NCT503 and their respective IC50s were calculated. After 96 h of incubation, cell proliferation was measured by the XTT assay method described below. Inhibition data were used to calculate IC50 values using nonlinear graphpad prism ver. 8.00 for Windows (GraphPad Software, San Diego, CA, USA).

### 2.4 Metabolomics analysis

Metabolomics analysis was performed at Human Metabolome Technologies (Tsuruoka, Japan, http://humanmetabolome.com). Cellular metabolites were extracted according to the manufacturer's protocol. Metabolomics analysis was performed by capillary electrophoresis time‐of‐flight mass spectrometry. Metabolite peaks were quantified and normalized to the protein concentration. For experiments using PHGDH inhibitors, cells were incubated with NCT503 adjusted to 30 μm and erdafitinib adjusted to 10 μm for 24 h, and the extracted products were submitted for evaluation. DMSO was used to dilute both inhibitors according to the manufacturer's protocol.

### 2.5 Western blotting

NuPAGE LDS Sample Buffer (Invitrogen; Thermo Fisher Scientific, Inc., Tokyo, Japan) was used to adjust the concentration of the total protein lysates. The following antibodies were used for immunoblotting: anti‐*PHGDH* (1 : 1000) (HPA021241; Sigma, St. Louis, MO, USA), anti‐*HIF1α* (1 : 1000; 2764; Cell Signaling Technologies, Inc., Danvers, MA, USA), anti‐IDH2 (1 : 1000; 12652; Cell Signaling Technologies), anti‐fatty acid synthase antibody (1 : 1000; C20G5; Cell Signaling Technologies), anti‐phospho‐*AKT* (1 : 1000; D9E; Cell Signaling Technologies), anti‐phospho‐p44/42 *MAPK* (Erk1/2) (1 : 1000; D13.14.4E; Cell Signaling Technologies), anti‐*BAX* (1 : 10 000; 50599‐2‐Ig; Proteintech Group, Inc., Chicago, IL, USA), anti‐*PHD‐2/Egln1* Antibody (1 : 1000; 3293; Cell Signaling Technologies) and anti‐β‐actin (1 : 5000; bs‐0061R; Bios, Beijing, China). The secondary antibody was peroxidase‐conjugated mouse anti‐rabbit IgG (1 : 5000; 7074S; Cell Signaling Technologies). Protein levels were assessed using imagej software (ver. 1.52; National Institutes of Health, Bethesda, MD, USA) as described previously [18].

### 2.6 siRNA transfection

Bladder cancer cells were transfected with 10 nm siRNA using Lipofectamine RNAiMAX transfection reagent (Thermo Fisher Scientific) and Opti‐MEM (Thermo Fisher Scientific), as reported previously Loss‐of‐function experiments were performed using siRNA targeting*PHGDH* (si‐*PHGDH*; cataloguenos. SASI_Hs01_00041882 and SASI_Hs01_00041884; Sigma) and negative control siRNA (D‐001810‐10; Dharmacon; Horizon Discovery Group, Cambridge, UK).

### 2.7 Cell proliferation, migration ability, invasion ability, and apoptosis assays

Cell proliferation was evaluated using the XTT assay and trypan blue exclusion assay. For the XTT assay, T24 and J82 cells, along with their gemcitabine‐resistant and cisplatin‐resistant cell lines, were seeded in 96‐well plates at 2000/well in 100 μL medium containing 10% FBS, and this seeding process was repeated six times. At 96 h after seeding, cell proliferation was measured using the Cell Proliferation Kit II (Roche Diagnostics GmbH, Mannheim, Germany) as described above. For the trypan blue exclusion assay, cells (2.0 × 10^5^/well) were seeded in 6‐well plates for 48 h and harvested by trypsinization. Harvested cells were resuspended in 50 μL PBS, and cell suspensions were diluted 1 : 1 with 0.4% trypan blue. After a 5‐min incubation, the cells were loaded onto a blood cell calculator, and viability was calculated by counting live (unstained) and dead (blue‐stained) cells using a microscope. This process was repeated three times. Cells were treated with 10 nm si‐*PHGDH*, 10 μm erdafitinib and 30 μm NCT503 at the same time.

A wound healing assay was used to evaluate cell migration. Cells (2.0 × 10^5^/well) were seeded in 6‐well plates and treated with 10 nm si‐*PHGDH*, 5 μm erdafitinib, and 30 μm NCT503 for 48 h. Then, a scratch was made in the resulting cell monolayer using a P‐1000 micropipette tip. The initial gap length at 0 h and the remaining gap length after 12 h were calculated from micrographs. Three random microscopic fields were used for quantification.

For the cell infiltration assay, Bio Coat Matrigel infiltration chambers coated with a thin layer of cell culture insert Matrigel basement membrane matrix with an 8.0‐μm‐pore‐size PET membrane were used in 24‐well tissue culture companion plates (Corning, Bedford, MA, USA). Cells (2.0 × 10^5^/well) were seeded into 6‐well plates and treated with 10 nm for si‐*PHGDH*, 10 μm erdafitinib, and 30 μm NCT503 for 48 h. After the cell counts were adjusted, cells that passed through the pores and adhered to the surface of the chamber after 24 h were counted from micrographs. Eight randomized microscopic fields were used for quantification.

For the apoptosis assay, cells (2 × 10^5^/well) were seeded in 6‐well plates and treated with 30 μm NCT503 and 10 μm erdafitinib. After 24 h, apoptosis was measured by flow cytometric determination using the CytoFLEX analyzer (Beckman Coulter, Brea, CA, USA) and FITC Annexin V Apoptosis Detection Kit (BD Biosciences) as per the manufacturer's recommendations. The positive control was 5 μg·mL^−1^ cycloheximide (Sigma). Each experiment was repeated at least three times.

### 2.8 Immunohistochemistry

Immunohistochemistry was performed using the UltraVision detection system (Thermo Scientific, Fremont, CA, USA) according to Thermo Scientific's protocol. A primary rabbit monoclonal antibody against *Ki67* (ab92742; Abcam, Cambridge, UK) was diluted 1 : 500 and incubated overnight at 4 °C. Then the cells were incubated with 5 μg·mL^−1^ of the secondary antibody, Goat Anti‐Rabbit IgG Antibody (H + L), Biotinylated (BA‐1000; Vector Laboratories, San Francisco, CA, USA) for 30 min. Positive cells were quantified by counting six random microscopic fields using a magnification of 200×.

### 2.9 *In silico* analysis

Data from The Cancer Genome Atlas (TCGA) cohort consisting of 436 patients with bladder urothelial carcinoma were used to assess the clinical relevance of our findings. This study followed the publication guidelines provided by TCGA. Kaplan–Meier analysis was used to analyze overall survival based on data from OncoLnc (http://www.oncolnc.org).

### 2.10 Statistical analysis

Differences between two groups were analyzed using the Mann–Whitney *U* test. Differences among three or more groups were analyzed using the Bonferroni/Dunn's multiple comparison test. All analyses were conducted using expert statview software, version 5.0 (SAS Institute, Inc., Cary, NC, USA).

### 2.11 Ethics approval and consent to participate

The protocol and study was approved by the Kagoshima University Animal Experiment Committee (MD23010), and the experiments were conducted in accordance with the Animal Use Consent Guidelines of the Kagoshima University Animal Care and Use Committee. Clinical data from the study patients were obtained from TCGA, a publicly available cancer genome database.

## 3 Results

### 3.1 Establishment of gemcitabine‐/cisplatin‐resistant BC mouse strains

**Fig. 1:**
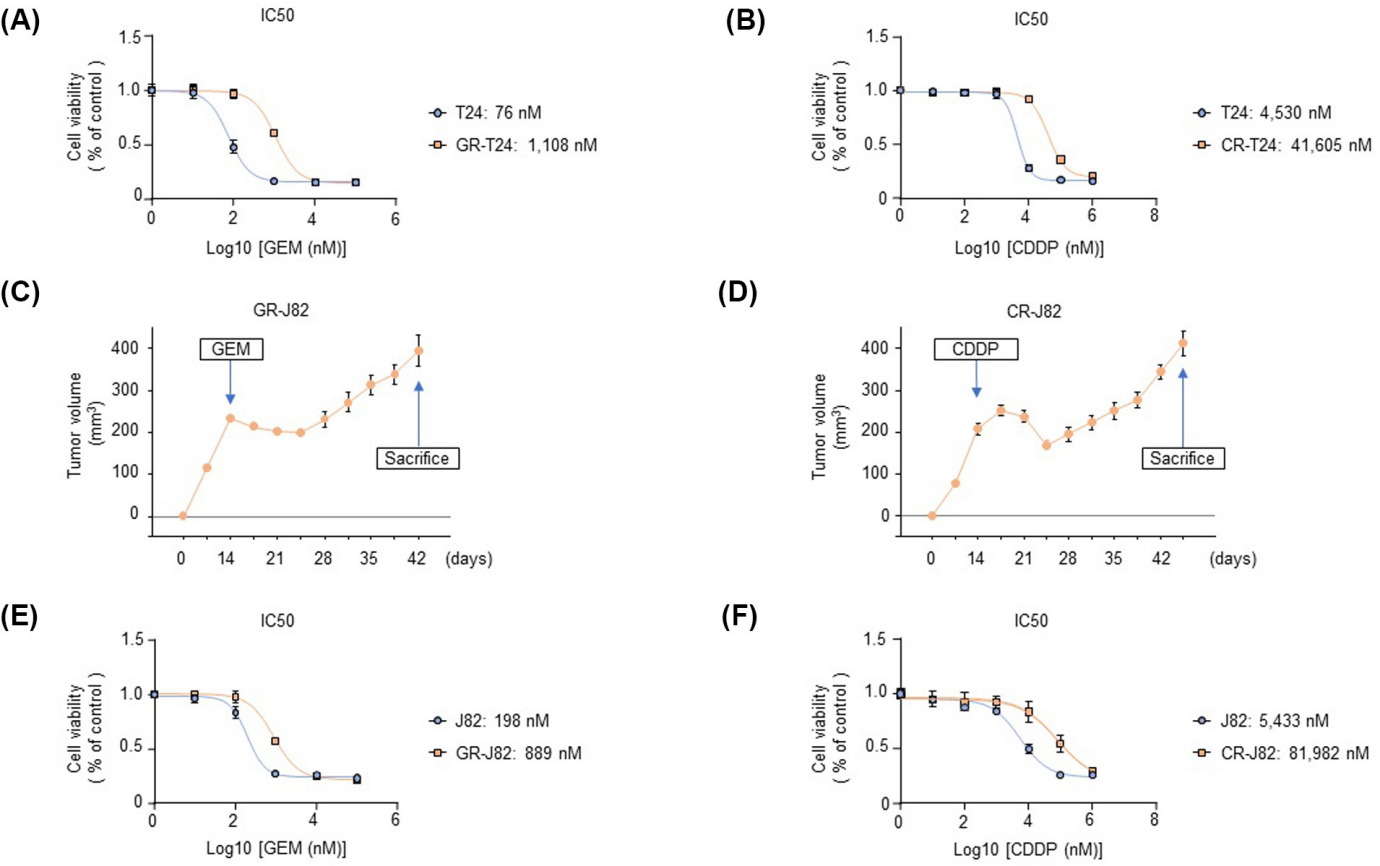
Establishment of gemcitabine‐ and cisplatin‐resistant cells (T24 and J82). (A) IC50 values of parental T24 and gemcitabine‐resistant T24 cells (*n* = 3). The error bars indicate SEM. (B) IC50 values of parental T24 and cisplatin‐resistant T24 cells (*n* = 3). The error bars indicate SEM. (C) Establishment of gemcitabine‐resistant J82 cells *in vivo*. The tumor volume in mice subcutaneously injected with parental J82 cells after treatment with gemcitabine (150 mg·kg^−1^, once a week) is shown (*n* = 3). The error bars indicate SEM. (D) Establishment of cisplatin‐resistant J82 cells *in vivo* (*n* = 3). The tumor volume in mice subcutaneously injected with parental J82 cells after treatment with cisplatin (4 mg·kg^−1^, five times a week) is shown (*n* = 3). The error bars indicate SEM. (E) IC50 values of parental J82 and gemcitabine‐resistant J82 cells treated with gemcitabine (*n* = 3). The error bars indicate SEM. (F) IC50 values of parental J82 and cisplatin‐resistant J82 cells treated with cisplatin (*n* = 3). The error bars indicate SEM. CDDP, cisplatin; CR‐J82, cisplatin‐resistant J82; CR‐T24, cisplatin‐resistant T24; GEM, gemcitabine; GR‐J82, gemcitabine‐resistant J82; GR‐T24, gemcitabine‐resistant T24; IC50, half maximal (50%) inhibitory concentration.

Gemcitabine‐resistant T24 and cisplatin‐resistant T24 cells previously established in our laboratory were continuously exposed to gemcitabine and cisplatin to maintain resistance. The IC50 was calculated to determine the resistance of the cells, and the IC50 was 14.6‐fold higher in GEM‐R‐T24 than in T24 cells (Fig. 1A). The IC50 concentration was 9.18‐fold higher in CDDP‐R‐T24 than in T24 cells (Fig. 1B).

We established GEM‐R‐J82 and CDDP‐R‐J82 *in vivo*. We subcutaneously inoculated J82 cells into nude mice and initiated the administration of gemcitabine and cisplatin on the 14th day. Tumor growth initially showed a reduction in response to chemotherapy; however, it gradually started to increase, indicating the development of resistance. At the point of acquiring resistance, the mice were sacrificed, and tumors were excised. From the excised cells, we established GEM‐R‐J82 and CDDP‐R‐J82, respectively (Fig. 1C,D). IC50 was calculated to evaluate the resistance of GEM‐R‐J82 and CDDP‐R‐J82 cells. The IC50 was 4.49 times higher in GEM‐R‐J82 than J82 cells (Fig. 1E). The IC50 was 15.09 times higher in CDDP‐R‐J82 than J82 cells (Fig. 1F). *In vitro* IC50 values provide initial insights into drug potency, but *in vivo* IC50 values are more relevant to understanding how a drug performs in a whole organism, which is critical for predicting clinical efficacy [22]. Animal experiments were approved by the Kagoshima University Animal Experiment Committee (MD22052) and conducted in accordance with the animal licensing guidelines of the Kagoshima University Animal Care and Control Committee.

### 3.2 Metabolomics analysis revealed an increase in aerobic glycolysis and PHGDH by stabilization of HIF1α in gemcitabine‐resistant cells

**Fig. 2:**
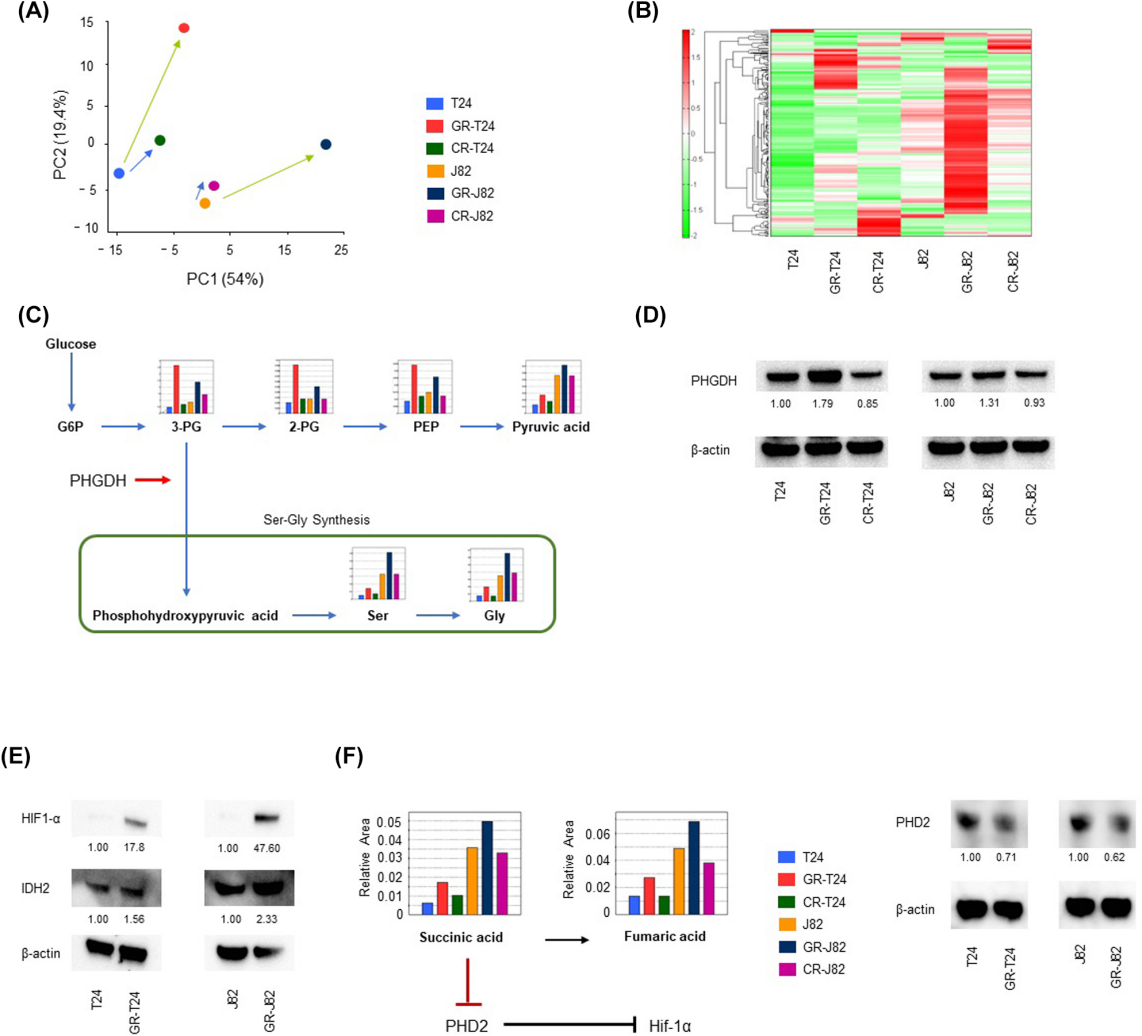
Increased aerobic glycolysis and PHGDH expression in gemcitabine‐resistant cells according to metabolomics analysis. (A) Principal component analysis of metabolites in parental T24, gemcitabine‐resistant T24, cisplatin‐resistant T24, parental J82, gemcitabine‐resistant J82, and cisplatin‐resistant J82 cells (*n* = 1). (B) Heatmap representation of the metabolites (*n* = 1). (C) Intracellular concentrations of major metabolites in glycolytic and serine biosynthesis pathways. Representative metabolites such as 3‐phosphoglycerin (3PG), 2‐phosphoglycerin (2PG), phosphoenolpyruvate (PEP), pyruvate, serine (Ser), and glycine (Gly) are shown. (D) Western blotting of PHGDH in parental T24, gemcitabine‐resistant T24, cisplatin‐resistant T24, parental J82, gemcitabine‐resistant J82, and cisplatin‐resistant J82 cells (*n* = 3). The expression of gemcitabine‐resistant cells was higher than parental cells. The number below each blot indicated PHGDH expression value normalized by β‐actin. (E) Expression of HIF1α and IDH2 in parental T24, gemcitabine‐resistant T24, parental J82, and gemcitabine‐resistant J82 cells analyzed by western blotting (*n* = 3). In gemcitabine‐resistant cells, an increase in the expression of HIF1α was observed due to the acquisition of function of IDH2. The number below each blot indicated HIF1α or IDH2 expression value normalized by β‐actin. (F) Metabolomics analysis of fumaric acid and succinic acid metabolites in parental and resistant cells. Expression of PHD2 in parental and gemcitabine‐resistant strains (*n* = 3). Gemcitabine‐resistant cells showed a decrease in PHD2 expression. These experiments were repeated at least three times. CR‐J82, cisplatin‐resistant J82; CR‐T24, cisplatin‐resistant T24; GR‐J82, gemcitabine‐resistant J82; GR‐T24, gemcitabine‐resistant T24. The number below each blot indicated IDH2 expression value normalized by β‐actin.

Metabolomics analysis showed clear changes in cell metabolite levels in the parental and gemcitabine‐resistant cells, and principal component analysis of cisplatin‐resistant cells showed clear changes in gemcitabine‐resistant cells (Fig. 2A). Changes in metabolites were observed between each pair of cell lines (Fig. 2B). Furthermore, pathway analysis showed that aerobic glycolysis and the synthesis of serine and glycine were enhanced in the gemcitabine‐resistant cells (Fig. 2C). In accordance with this, western blotting also showed increased expression of *PHGDH* in the gemcitabine‐resistant cells (Fig. 2D).

Shigeta et al. [23] reported that gemcitabine‐resistant isocitrate dehydrogenase 2 (IDH2) induces reductive glutamine metabolism, stabilizes *HIF1α*, and enhances aerobic glycolysis. Also, western blot analysis confirmed the increased expression of *HIF1α* and *IDH2* (Fig. 2E) and reductive glutamine metabolism (Fig. S1). Metabolomics analysis showed that succinic acid and fumaric acid levels increased in the gemcitabine‐resistant cells, suggesting that PHD2 might be suppressed in this strain (Fig. 2F).

### 3.3 Knockdown of PHGDH in parental, gemcitabine‐resistant, and cisplatin‐resistant BC cells suppressed cell proliferation, migration, and invasion

**Fig. 3:**
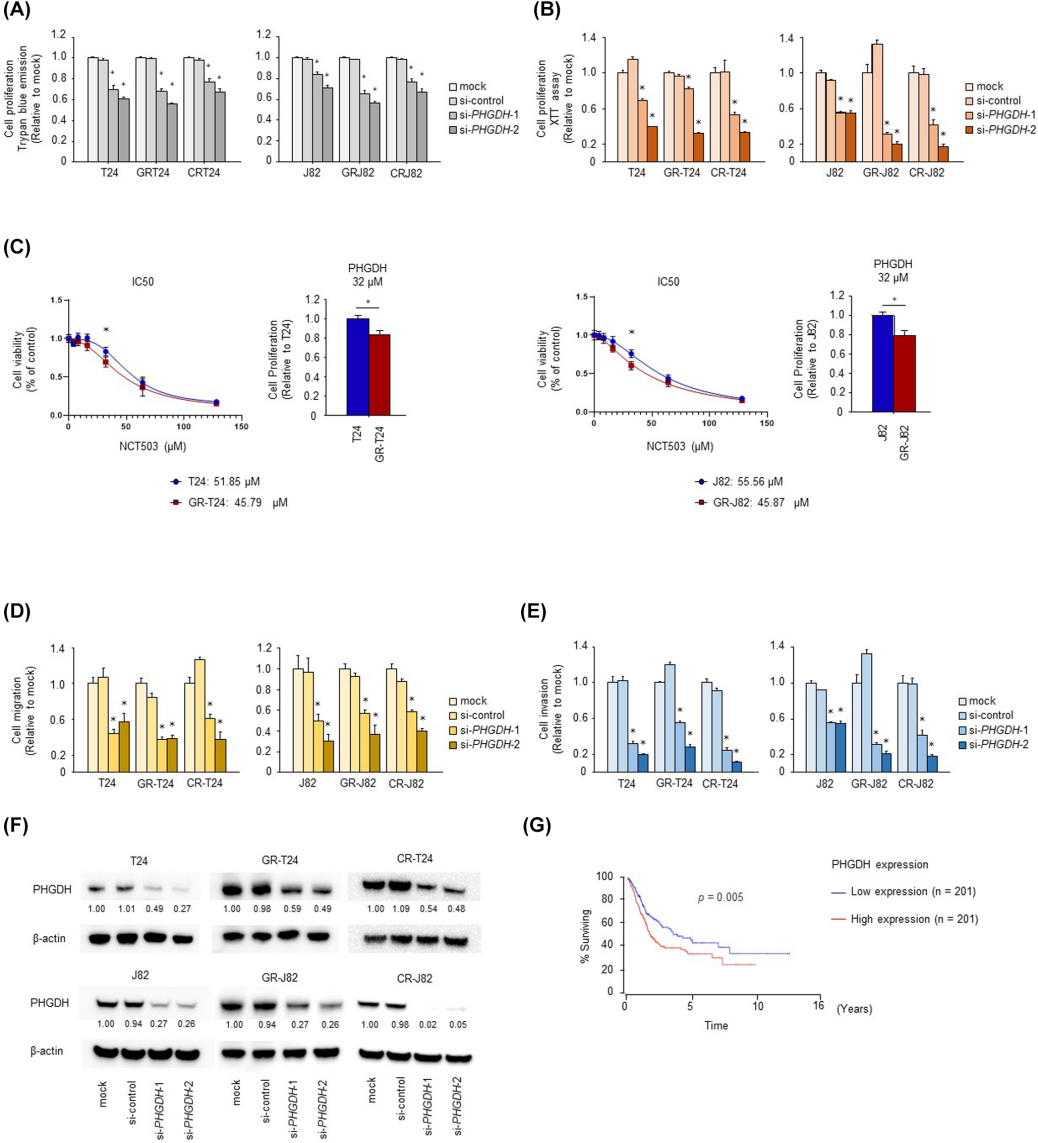
Loss‐of‐function assays showed downregulated PHGDH in drug‐resistant bladder cancer cells. (A, B) Cell proliferation according to trypan blue exclusion assay and XTT assay, after si‐PHGDH transfection (*n* = 6, *P < 0.083, Bonferroni/Dunn's multiple comparison test). The error bars indicate SEM. (C) IC50 of NCT503 for parental and gemcitabine‐resistant cells (*n* = 3, *P < 0.05, Mann–Whitney *U* test). The error bars indicate SEM (D) Cell migration activity according to the wound healing assay (*n* = 3, *P < 0.083, Bonferroni/Dunn's multiple comparison test). The error bars indicate SEM. (E) Cell invasion activity according to the Matrigel invasion assay (*n* = 8, *P < 0.083, Bonferroni/Dunn's multiple comparison test). The error bars indicate SEM. (F) Expression of PHGDH according to western blotting in si‐PHGDH‐transfected cells (*n* = 3). (G) Kaplan–Meier survival curves using the OncoLnc data set showing significantly shorter overall survival in the high than low PHGDH expression group (*P* = 0.005, Mann–Whitney *U* test). These experiments were repeated at least three times. CR‐J82, cisplatin‐resistant J82; CR‐T24, cisplatin‐resistant T24; GR‐J82, gemcitabine‐resistant J82; GR‐T24, gemcitabine‐resistant T24.

Because PHGDH expression was elevated in gemcitabine‐resistant BC cells, we performed a PHGDH loss‐of‐function assay. Cell proliferation, according to trypan blue exclusion assay and XTT assay, of si‐PHGDH‐transfected parental cells (T24 and J82) and resistant cell lines (GEM‐R‐T24, GEM‐R‐J82, CDDP‐R‐T24, and CDDP‐R‐J82) was significantly inhibited compared with control cells (mock and control siRNA) (Fig. 3A,B). The sensitivity to NCT503 which is a PHGDH inhibitor was significantly improved at 32 μm in gemcitabine‐resistant cells, where PHGDH expression was elevated (Fig. 3C). Furthermore, cell migration, according to the wound healing assay, and cell invasion, according to the Matrigel invasion assay, were also significantly inhibited in si‐PHGDH‐transfected cells compared with the control cells (mock and control siRNA) (Fig. 3D,E; Figs S2–S4). si‐PHGDH was transfected into the cells, and western blotting confirmed suppression of PHGDH expression (Fig. 3F). Analysis using the OncoLnc dataset showed that high PHGDH expression is a poor prognostic factor for overall survival (*P* < 0.0001; Fig. 3G). TCGA cohort tended to have a high degree of pathology in the PHGDH high expression group, and there were many stages greater than cT stage T3 (Fig. S5).

### 3.4 Fatty acid synthesis was enhanced, and proliferation, migration, and invasion were significantly suppressed, by erdafitinib and NCT503 combination therapy in cisplatin‐resistant BC cells

**Fig. 4:**
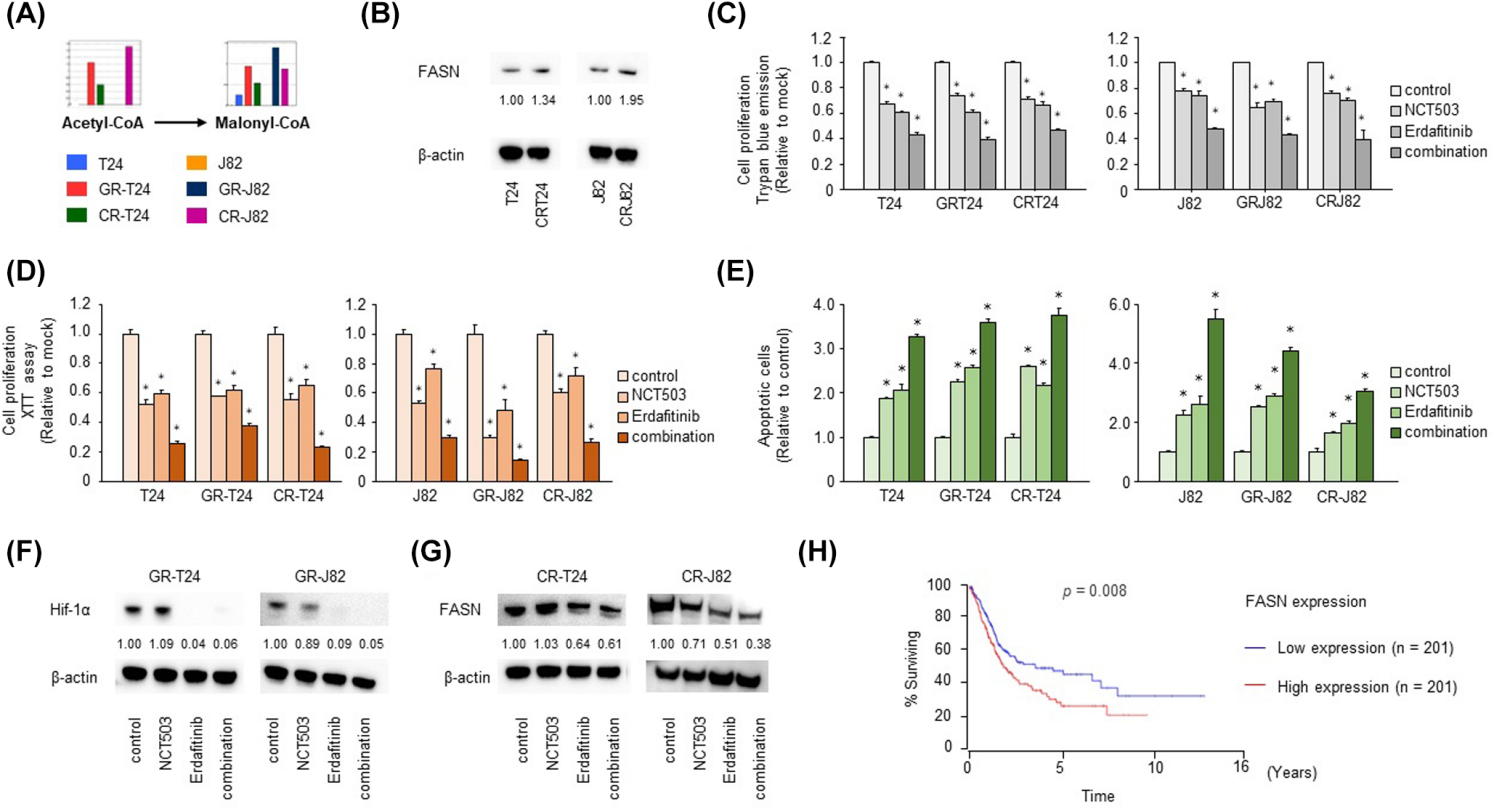
Elevated levels of fatty acid metabolites in cisplatin‐resistant cells and loss‐of‐function assays using the PHGDH inhibitor NCT503 combined with erdafitinib. (A) Intracellular concentrations of major metabolites of fatty acid metabolism according to metabolomics analysis. Acetyl‐CoA and malonyl‐CoA are shown as representative glycolytic metabolites (*n* = 1). (B) Upregulated FASN expression in parental T24, cisplatin‐resistant T24, parental J82, and cisplatin‐resistant J82 cells according to western blotting. Increased expression of FASN was observed in cisplatin‐resistant cells (*n* = 1). The numbers below the blot indicate intensity. (C, D) Cell proliferation according to trypan blue exclusion assay and XTT assay after NCT503 plus erdafitinib combination treatment (*n* = 6, *P < 0.083, Bonferroni/Dunn's multiple comparison test). The error bars indicate SEM. (E) Apoptosis assay using flow cytometric analysis (*n* = 3, *P < 0.0083, Bonferroni/Dunn's multiple comparison test). The error bars indicate SEM. (F) HIF1α expression in gemcitabine‐resistant cells after combination therapy according to western blotting (*n* = 3). (G) Downregulation of FASN expression in cisplatin‐resistant cells after combination therapy according to western blotting (*n* = 1). (H) Survival curves for the high and low FASN expression groups using OncoLnc (*P* = 0.008, Mann–Whitney *U* test). These experiments were repeated at least three times. CR‐J82, cisplatin‐resistant J82; CR‐T24, cisplatin‐resistant T24; GR‐J82, gemcitabine‐resistant J82; GR‐T24, gemcitabine‐resistant T24.

Metabolomics analysis revealed elevated levels of acetyl‐CoA and malonyl‐CoA, involved in fatty acid synthesis, in cisplatin‐resistant BC cells (Fig. 4A), and the expression of fatty acid synthase (*FASN*), downstream of cisplatin‐resistant malonyl‐CoA, was also increased, according to western blotting (Fig. 4B). *EGFR*, phosphatidylinositol 3‐kinase (*PI3K*)/*AKT*, and *RAS/MAPK* signaling pathways are important regulators of metabolism, including lipid metabolism (such as that involving *FASN*), in cancer [24]. Considering that *FGFR* may regulate lipids as well, we performed a loss‐of‐function assay using NCT503 combined with erdafitinib, an *FGFR* inhibitor used as a second‐line therapy for invasive BC. The combination of erdafitinib and NCT503 additively suppressed proliferation in the trypan blue exclusion assay and XTT assay (Fig. 4C,D), cell migration in the wound healing assay, and cell invasion in the Matrigel invasion assay (Figs S6A,B and S7–S9). An apoptosis assay showed that the combination of erdafitinib and NCT503 predominantly induced apoptosis (Fig. 4E; Fig. S10). Western blotting showed that the combination of erdafitinib and NCT503 increased the level of *BAX*, a pro‐apoptotic protein (Fig. S11A). HIF1α protein synthesis is regulated by activation of the *PI3K* and *ERK* pathways, which are activated by signaling via tyrosine kinase receptors, non‐tyrosine kinase receptors, and G protein‐coupled receptors [25]. Erdafitinib, an inhibitor of the tyrosine kinase receptor FGFR, was used to suppress HIF1α, which was elevated in gemcitabine‐resistant cells, as confirmed by western blotting (Fig. 4F). It was inferred that inhibition of *FGFR* suppressed *p‐AKT* and *p‐ERK* and the downstream *HIF1α* (Fig. S11B). Erdafitinib also significantly suppressed FASN in cisplatin‐resistant cells (Fig. 4G). High expression of *FASN* is a poor prognostic factor in BC according to the OncoLnc database (Fig. 4H).

### 3.5 *In vivo* treatment with NCT503 plus erdafitinib reduced tumor size. Metabolomics analysis showed that the combination of erdafitinib and NCT503 suppressed tumor metabolism primarily by inhibiting phosphoribosyl pyrophosphate formation, nucleotide synthesis, and the fatty acid precursor malonyl‐CoA in the pentose phosphate pathway

**Fig. 5:**
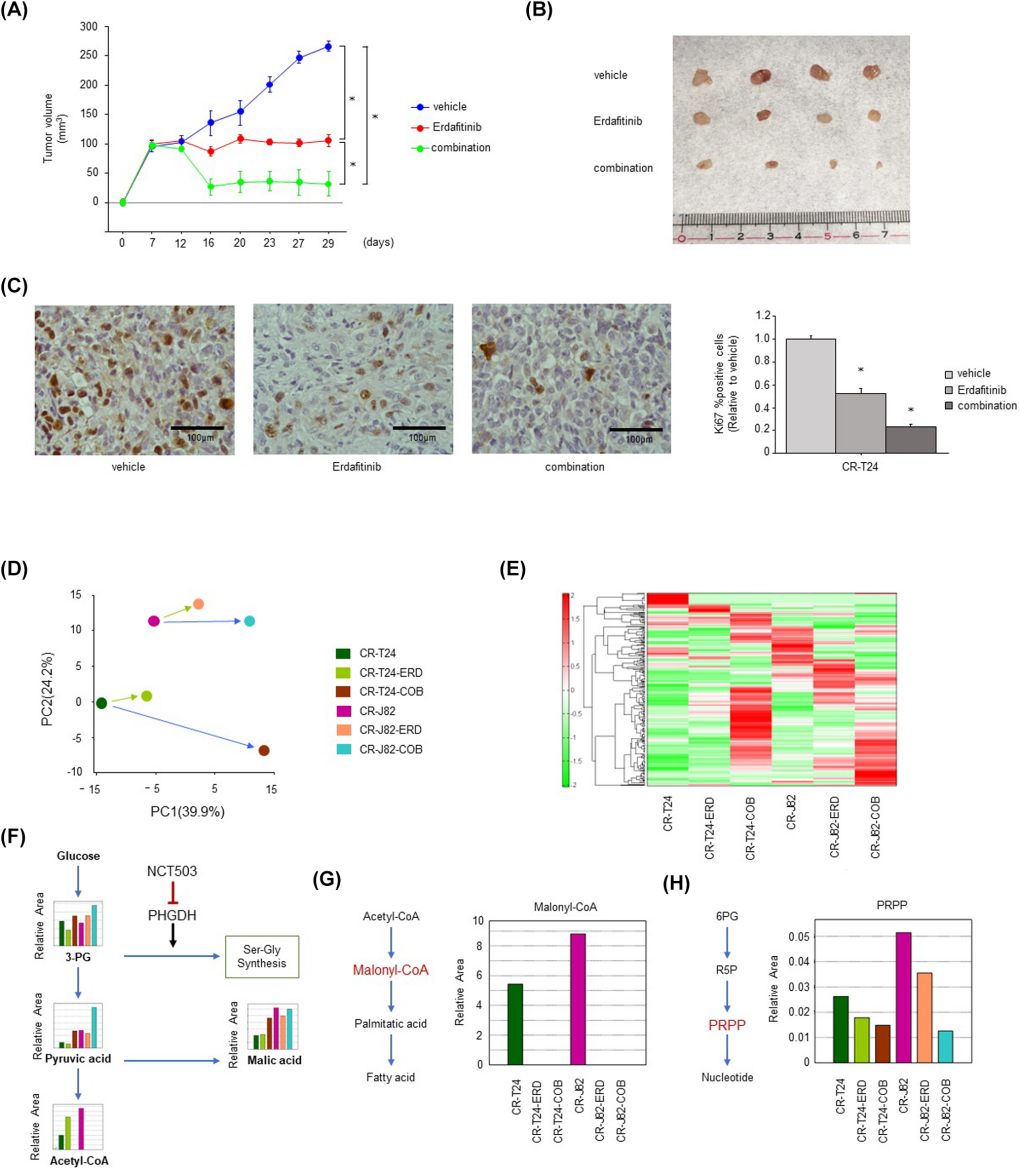
Combination NCT503 and erdafitinib therapy in a cisplatin‐resistant T24 xenograft mouse model. (A) Change in tumor volume over time (*n* = 4, *P < 0.0083, Bonferroni/Dunn's multiple comparison test). The error bars indicate SEM. (B) Images of the tumors (*n* = 4). (C) Rate of Ki67‐positive cells in the immunostained tissues samples (*n* = 8, *P < 0.0167, Bonferroni/Dunn's multiple comparison test). Scale bar, 100 μm. The error bars indicate SEM. Metabolomics analysis using the PHGDH inhibitor NCT503 combined with erdafitinib. (D) Principal component analysis of intracellular metabolites in cisplatin‐resistant T24/J82 cells after erdafitinib alone or combination therapy (*n* = 1). (E) Heat map of the metabolites (*n* = 1). (F) Intracellular concentrations of the key metabolites in the glycolytic and serine–malate biosynthesis pathways (*n* = 1). (G) Fatty acid synthesis from acetyl‐CoA via malonyl‐CoA (*n* = 1). (H) Intracellular concentrations of the major metabolites of the nucleotide synthesis pathway in the pentose phosphate pathway (*n* = 1). COB, combination; CR‐J82, cisplatin‐resistant J82; CR‐T24, cisplatin‐resistant T24; ERD, erdafitinib; GR‐J82, gemcitabine‐resistant J82; GR‐T24, gemcitabine‐resistant T24.

Tumors were xenografted into three mouse treatment groups: the vehicle group, erdafitinib alone group, and erdafitinib plus NCT503 group. One week after Xenograft, Veichle, Erdafitinib, and Erdafitinib + NCT503 were administered. It was sacrificed 29 days after xenograft. The change in tumor diameter was assessed (Fig. 5A), and the tumor diameter was significantly smaller in the erdafitinib plus NCT503 group. Tumor was significantly suppressed in the removed specimen (Fig. 5B). There was no difference in body weight among the groups (Fig. S12). Immunostaining of excised tissue showed a significant decrease in Ki67 expression, indicating reduced cell proliferation (Fig. 5C). Tumors were also xenografted into two additional mouse treatment groups: a vehicle group and an NCT503 alone group. One week after xenotransplantation, vehicle and NCT503 were administered. Mice were sacrificed 25 days after xenografting. Changes in tumor diameter were evaluated (Fig. S13A), and tumor diameter was significantly smaller in the erdafitinib + NCT503 group compared with the vehicle group. Tumor growth was significantly suppressed in the excised specimens (Fig. S13B). There was no difference in body weight between groups (Fig. S13C). Metabolomics analysis showed clear changes in cellular metabolites in the parental cells, erdafitinib alone treatment group, and erdafitinib plus NCT503 treatment group (Fig. 5D,E). NCT503 decreased the synthesis of glucose‐derived acetyl‐CoA and increased the conversion of glucose‐derived carbon from pyruvate to malate (Fig. 5F). Erdafitinib inhibited fatty acid synthesis via malonyl‐CoA (Fig. 5G). The combination of erdafitinib plus NCT503 also induced predominant downregulation of phosphoribosyl pyrophosphate (PRPP) synthesis and nucleotide formation by the pentose phosphate pathway (Fig. 5H).

### 3.6 Gemcitabine‐resistant cells showed increased aerobic glycolysis, while cisplatin‐resistant cells showed increased fatty acid synthesis

**Fig. 6:**
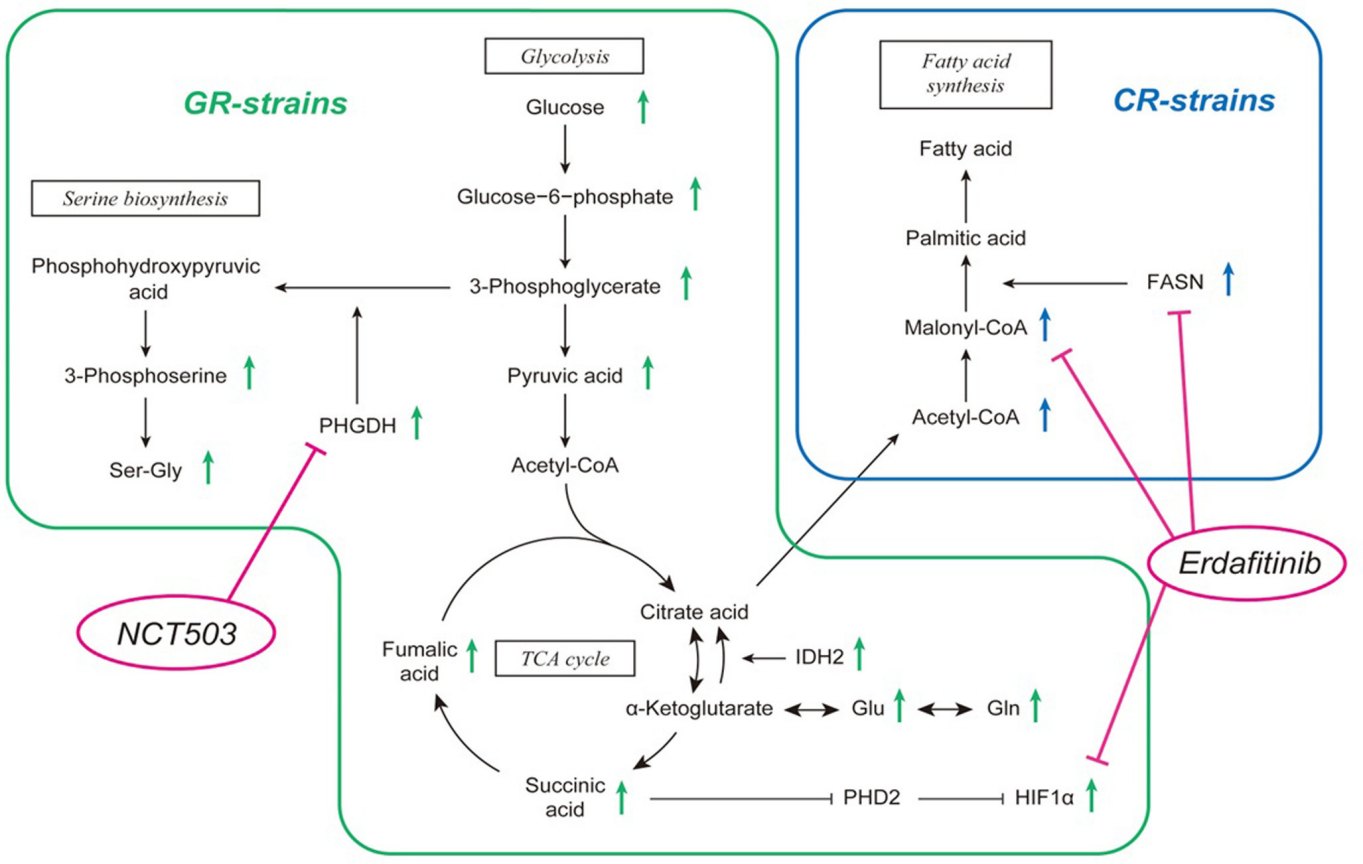
Schematic diagram of drug‐resistant bladder cancer treated with NCT503 plus Erdafitinib combination therapy. PHGDH and HIF1α, which are elevated in gemcitabine‐resistant cells, were suppressed by NCT503 and Erdafitinib. FASN, which is elevated in cisplatin‐resistant cells, was well suppressed by Erdafitinib. CR‐strains, cisplatin‐resistant strains; GR‐strains, gemcitabine‐resistant strains; TCA, citric acid cycle.

Gemcitabine‐resistant cells showed increased PHGDH and HIF1α, which were suppressed by NCT503 and Erdafitinib. In cisplatin‐resistant cells, fatty acid synthesis was enhanced, and erdafitinib suppressed FASN and malonyl‐CoA levels (Fig. 6).

## 4 Discussion

Alterations in cellular metabolism are important features of cancer cells, inducing uncontrolled growth, evasion of cell death, and metastasis [26]. Cellular metabolic reprogramming in cancer is regulated by several oncogenic proteins such as HIF1, c‐myc, p53, *PI3K/Akt/mTOR* pathway members, and tumor suppressors [27]. This has recently led to studies evaluating therapies that target metabolism [24, 27]. Gemcitabine is an antimetabolite, a drug that interferes with DNA replication and hinders tumor growth. Regarding gemcitabine resistance, it was reported that HIF1α is stabilized by increased expression of MUC1 in pancreatic cancer [28]. As for BC, Shigeta et al. [23] reported that gain of function of *IDH2* enhanced aerobic glycolysis in gemcitabine‐resistant BC by inducing glutamine metabolism and *HIF1α* expression. This report is consistent with our results. In this study, metabolomic analysis showed that the synthesis of serine and glycine is upregulated in glucose metabolism, which is inferred to be due to increased expression of *PHGDH*. Succinic acid has been reported to stabilize HIF1α by inhibiting *PHD2*, which is required for HIF1α degradation via the ubiquitin proteasome system [29, 30, 31], and this was observed in gemcitabine‐resistant cells. On the other hand, our result that the increased levels of succinate and fumarate were correlated with HIF1α upregulation has not been demonstrated, therefore increased succinate and fumarate may not directly cause HIF1α upregulation. *PHGDH* is a key enzyme in serine synthesis and is involved in the synthesis of NADPH and glycine. Activation of serine biosynthesis contributes to cancer cell proliferation, and overexpression of *PHGDH* has been observed in various cancers [21, 32, 33, 34, 35]. Glycine is necessary for the synthesis of glutathione, which is essential for tumorigenesis [36]. Previous studies have reported that NCT503, a small molecule *PHGDH* inhibitor, impairs the synthesis of glucose‐derived serine and induces apoptosis in BC, thereby suppressing tumor growth [37]. High *PHGDH* expression is a poor prognostic factor for BC [37]. High PHGDH expression has also been reported as a poor prognostic factor in patients with advanced or recurrent non‐small cell lung cancer treated with anti‐PD‐1/PD‐L1 antibodies, which would suggest that *PHGDH* inhibitors have potential clinical application [38]. Because there have been no clinical trials of *PHGDH* inhibitors, a trial is needed in the near future. On the other hand, higher expression of HIF1α has been shown in various cancers to indicate drug resistance [39, 40]. Semenza [25] reported that protein synthesis of *HIF1α* is regulated by the upstream *AKT* and *ERK* signaling pathways and can be activated by signaling through tyrosine kinase receptors. To the best of our knowledge, this is the first evidence that HIF1α, which is elevated by gemcitabine resistance, is suppressed by erdafitinib.

Cisplatin is a platinum‐based anticancer drug that destroys cancer cells by interfering with DNA replication. The mechanism of acquiring resistance to cisplatin is very complex. It has been reported that fatty acid synthesis may enable plasma membrane remodeling by altering fatty acid and lipid compositions [41, 42]. Fatty acids are synthesized in the cytosol from acetyl‐CoA, which is generated from the breakdown of citrate via ATP citrate lyase. Acetyl‐CoA is then carboxylated into malonyl‐CoA via acetyl‐CoA carboxylase, and malonyl‐CoA is then converted to the 16‐carbon‐long fatty acid palmitic acid by the enzyme *FASN*. Enzymes involved in fatty acid synthesis are highly expressed in many types of cancer, and their pharmacological inhibition has been shown to exert anticancer activity [43]. ATP citrate lyase and *FASN* upregulation has been shown in colorectal, gastric, liver, and lung cancer, and their overexpression has been significantly associated with poor survival in lung cancer patients [44, 45]. In this study, metabolomics analysis of cisplatin‐resistant cells revealed elevated expression of acetyl‐CoA and malonyl‐CoA, which are involved in fatty acid synthesis, and confirmed the elevation of FASN, a related enzyme. High expression of *FASN* is also a poor prognostic factor for BC according to the OncoLnc database. Therapies that inhibit lipid metabolic pathways include those targeting acetyl‐CoA carboxylase in non‐small cell lung cancer and hepatocellular carcinoma as well as those targeting *FASN* in lung [46, 47], ovarian, and prostate cancers [48].

On the other hand, there are reports that EGFR, *PI3K/AKT*, and *RAS/MAPK* signaling pathways are key regulators of metabolism, including lipid metabolism, in cancer. PI3K/AKT, as well as RAS/MAPK, pathways modulate the expression of genes involved in fatty acid synthesis, such as *FASN* [24]. In this study, erdafitinib significantly suppressed the elevated levels of acetyl‐CoA and malonyl‐CoA, which are involved in fatty acid synthesis, in cisplatin‐resistant cells as revealed by metabolomics analysis. It also suppressed *FASN*, downstream of acetyl‐CoA and malonyl‐CoA. These results suggest that *FASN*, involved in fatty acid synthesis, is downstream of *FGFR*. As described above, we explored the mechanisms of resistance to Gemcitabine resistance and cisplatin resistance from a metabolic point of view, but in actual clinical practice, resistance to each drug is acquired from gemcitabine plus cisplatin combination therapy; therefore, in this study, we used a *PHGDH* inhibitor combined with erdafitinib.

Erdafitinib has been reported to inhibit the expression of c‐Myc and induce apoptosis via oxidative stress [49]. Additionally, there are reports suggesting that inhibiting fatty acid synthesis can induce apoptosis in hepatocellular carcinoma via the β‐catenin/c‐myc signaling pathway [50]. On the other hand, NCT503 may induce apoptosis by depletion of α‐ketoglutaric acid and augmentation of Reactive Oxygen Species [34]. In this study, we demonstrated that this combination therapy synergistically induces apoptosis and cell proliferation both *in vitro* and *in vivo*. Erdafitinib suppresses fatty acid synthesis, and it has been speculated that erdafitinib combined with NCT503 synergistically induce apoptosis, contributing to tumor shrinkage. Additionally, erdafitinib significantly suppresses PRPP, a key component in nucleotide synthesis. PRPP synthetase plays a regulatory role in cancer metabolism and promotes nucleotide synthesis under tumor stress conditions [51]. In prostate cancer, the inhibition of PRPP synthetase 2 has been reported to suppress the cell cycle and induce apoptosis [52]. Therefore, it was suggested that cooperative inhibition of PRPP might synergistically induce apoptosis. As a limitation, no studies have been conducted in gemcitabine‐ and cisplatin‐resistant BC, and this is a topic for future research. Therefore, further studies are needed to elucidate the roles of pan‐Ras inhibitor 3144 *in vitro* and *in vivo*.

## 5 Conclusion

In this study, we revealed the complex interplay of metabolic reprogramming in cancer cells and highlighted the therapeutic potential. By targeting key players in the metabolic network, including *PHGDH*, *FASN*, and *HIF1α*, we have paved the way for a deeper understanding of drug‐resistant BC. Employing a synergistic drug approach using NCT503 and erdafitinib has further illuminated this understanding in the context of metabolic alterations. These findings hold promise for the development of innovative strategies firmly rooted in the realm of metabolic reprogramming to combat this challenging disease.

## Conflict of interest

The authors declare no conflict of interest.

## Author contributions

HY and HE conceived and designed the project. IK, AM, TS, SI, RM, MN, ST, and YY acquired the data. IK, WF, JA, SS, GL, and IF analyzed the data. HY and IK wrote the paper. All authors reviewed the paper.

### Peer review

The peer review history for this article is available at https://www.webofscience.com/api/gateway/wos/peer‐review/10.1002/1878‐0261.13684.

## Supporting information


**Fig. S1.** Pathway analysis of changes in glucose metabolism in parental and resistant bladder cancer cells.
**Fig. S2.** Image of migration assay in parental and gemcitabine‐/cisplatin‐resistant T24 cells after downregulation of PHGDH.
**Fig. S3.** Image of migration assay in parental and gemcitabine‐/cisplatin‐resistant J82 cells after downregulation of PHGDH.
**Fig. S4.** Image of cell invasion assay in drug‐resistant bladder cancer cells after downregulation of PHGDH.
**Fig. S5.** Malignancy and T stage according to PHGDH expression using TCGA data.
**Fig. S6.** Migration and invasion assay in parental and gemcitabine‐/cisplatin‐resistant cells with combination NCT503 and erdafitinib therapy.
**Fig. S7.** Image of migration assay in parental and gemcitabine‐/cisplatin‐resistant T24 cells treated with combination NCT503 and erdafitinib therapy.
**Fig. S8.** Image of migration assay in parental and gemcitabine‐/cisplatin‐resistant J82 cells treated with combination NCT503 and erdafitinib therapy.
**Fig. S9.** Image of invasion assay of parental and resistant cell lines after NCT503 plus erdafitinib combination treatment.
**Fig. S10.** Apoptosis assay of parental and resistant cell lines after NCT503 plus erdafitinib combination treatment.
**Fig. S11.** Western blotting of BAX, p‐Erk, and p‐AKT after NCT503 and erdafitinib therapy.
**Fig. S12.** Body weight changes in mice treated with combination NCT503 and erdafitinib.
**Fig. S13.** Vehicle and NCT503 therapy in a cisplatin‐resistant T24 xenograft mouse model.

## Data Availability

The data that support the findings of this study are openly available in figshare. The reference numbers are as follows https://doi.org/10.6084/m9.figshare.24416800.
