# Supplementary material for: Targeting metabolic reprogramming to overcome drug resistance in advanced bladder cancer: insights from gemcitabine‐ and cisplatin‐resistant models
Source: Mol Oncol. 2024 Jun 14;18(9):2196–211. doi: 10.1002/1878-0261.13684 (PMC11467791; doi:10.1002/1878-0261.13684)
Supplement: Supplementary file 1 — Fig. S1. Pathway analysis of changes in glucose metabolism in parental and resistant bladder cancer cells. Fig. S2. Image of migration assay in parental and gemcitabine‐/cisplatin‐resistant T24 cells after downregulation of PHGDH. Fig. S3. Image of migration assay in parental and gemcitabine‐/cisplatin‐resistant J82 cells after downregulation of PHGDH. Fig. S4. Image of cell invasion assay in drug‐resistant bladder cancer cells after downregulation of PHGDH. Fig. S5. Malignancy and T stage according to PHGDH expression using TCGA data. Fig. S6. Migration and invasion assay in parental and gemcitabine‐/cisplatin‐resistant cells with combination NCT503 and erdafitinib therapy. Fig. S7. Image of migration assay in parental and gemcitabine‐/cisplatin‐resistant T24 cells treated with combination NCT503 and erdafitinib therapy. Fig. S8. Image of migration assay in parental and gemcitabine‐/cisplatin‐resistant J82 cells treated with combination NCT503 and erdafitinib therapy. Fig. S9. Image of invasion assay of parental and resistant cell lines after NCT503 plus erdafitinib combination treatment. Fig. S10. Apoptosis assay of parental and resistant cell lines after NCT503 plus erdafitinib combination treatment. Fig. S11. Western blotting of BAX, p‐Erk, and p‐AKT after NCT503 and erdafitinib therapy. Fig. S12. Body weight changes in mice treated with combination NCT503 and erdafitinib. Fig. S13. Vehicle and NCT503 therapy in a cisplatin‐resistant T24 xenograft mouse model. [file MOL2-18-2196-s001.zip › Supplement_FiguresS1-S13_legend.docx]

Supplementary Figure 1

Schematic diagram showing glucose metabolism of parental and gemcitabine/cisplatin-resistant cells by metabolomics analysis.

Aerobic glycolysis was enhanced in the gemcitabine-resistant cells.

Supplementary Figure 2

Representative pictures of si-PHGDH knockdown in cell migration assay of parental and gemcitabine/cisplatin-resistant T24 cells. Scale bar, 300 μm.

Supplementary Figure 3

Representative pictures of si-PHGDH knockdown in cell migration assay of parental and gemcitabine/cisplatin-resistant J82 cells. Scale bar, 300 μm.

Supplementary Figure 4

Representative pictures of si-PHGDH knockdown in cell invasion assay of parental and gemcitabine/cisplatin-resistant T24 and J82 cells. Scale bar, 100 μm.

Supplementary Figure 5

Using the TCGA cohort, PHGDH expression was shown in terms of pathological grade and clinical T-stage.

Supplementary Figure 6

NCT503 and Erdafitinib combination therapy was performed in parental and gemcitabine/cisplatin resistant strains and evaluated by migration assay and invasive assay.

1. Diagram of migration assay. (B) Diagram of invasion assay.

Supplementary Figure 7

Representative pictures of migration assay of NCT503 and Erdafitinib combination therapy in parental and gemcitabine/cisplatin resistant T24 cells. Scale bar, 300 μm.

Supplementary Figure 8

Representative pictures of migration assay of NCT503 and Erdafitinib combination therapy in parental and gemcitabine/cisplatin resistant J82 cells. Scale bar, 300 μm.

Supplementary Figure 9

Representative pictures of invasion assay of NCT503 and Erdafitinib combination therapy in parental and gemcitabine/cisplatin resistant T24 and J82 cells. Scale bar, 100 μm.

Supplementary Figure 10

Representative pictures of apoptosis assay of NCT503 and Erdafitinib combination therapy in parental and gemcitabine/cisplatin resistant T24 and J82 cells.

Supplementary Figure 11

Western blotting of apoptosis-related genes in combination therapy with NCT503 and Erdafitinib.

(A)Western blotting showed BAX expression in parental and gemcitabine/cisplatin resistant cells treated with NCT503 and Erdafitinib. (B) Western blotting showed p-AKR and p-ERK expression in parental and gemcitabine resistant cells treated with NCT503 and Erdafitinib.

Supplementary Figure 12

The figure shows the weight change of the vehicle, Erdafitinib, and Erdafitinib plus NCT503 groups after Xenografting of CRT-24.

Supplementary Figure 13

Comparison of CRT-24 with Xenografted, Vehicle, and NCT503 treatment groups. (A) Tumor volume in vehicle and NCT503 treatment groups. (B) Photographs of actual tumors in the Vehicle and NCT503 treatment groups. (C) Weight change in the Vehicle and NCT503 treatment groups.
