# Supplementary figures and images for: Targeting metabolic reprogramming to overcome drug resistance in advanced bladder cancer: insights from gemcitabine‐ and cisplatin‐resistant models
Source: Mol Oncol. 2024 Jun 14;18(9):2196–211. doi: 10.1002/1878-0261.13684 (PMC11467791; doi:10.1002/1878-0261.13684)

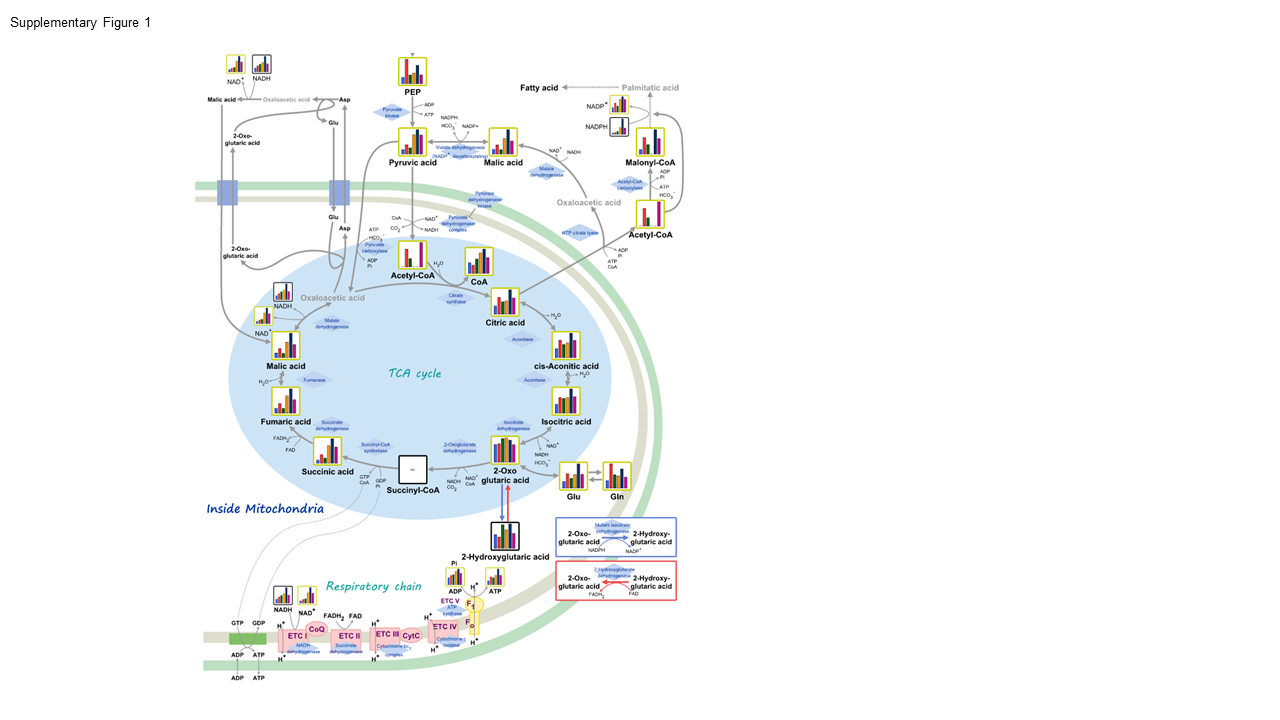

Supplement: Supplementary file 1 — Fig. S1. Pathway analysis of changes in glucose metabolism in parental and resistant bladder cancer cells. Fig. S2. Image of migration assay in parental and gemcitabine‐/cisplatin‐resistant T24 cells after downregulation of PHGDH. Fig. S3. Image of migration assay in parental and gemcitabine‐/cisplatin‐resistant J82 cells after downregulation of PHGDH. Fig. S4. Image of cell invasion assay in drug‐resistant bladder cancer cells after downregulation of PHGDH. Fig. S5. Malignancy and T stage according to PHGDH expression using TCGA data. Fig. S6. Migration and invasion assay in parental and gemcitabine‐/cisplatin‐resistant cells with combination NCT503 and erdafitinib therapy. Fig. S7. Image of migration assay in parental and gemcitabine‐/cisplatin‐resistant T24 cells treated with combination NCT503 and erdafitinib therapy. Fig. S8. Image of migration assay in parental and gemcitabine‐/cisplatin‐resistant J82 cells treated with combination NCT503 and erdafitinib therapy. Fig. S9. Image of invasion assay of parental and resistant cell lines after NCT503 plus erdafitinib combination treatment. Fig. S10. Apoptosis assay of parental and resistant cell lines after NCT503 plus erdafitinib combination treatment. Fig. S11. Western blotting of BAX, p‐Erk, and p‐AKT after NCT503 and erdafitinib therapy. Fig. S12. Body weight changes in mice treated with combination NCT503 and erdafitinib. Fig. S13. Vehicle and NCT503 therapy in a cisplatin‐resistant T24 xenograft mouse model. [file MOL2-18-2196-s001.zip › mol213684-sup-0001-FigS1.TIF]

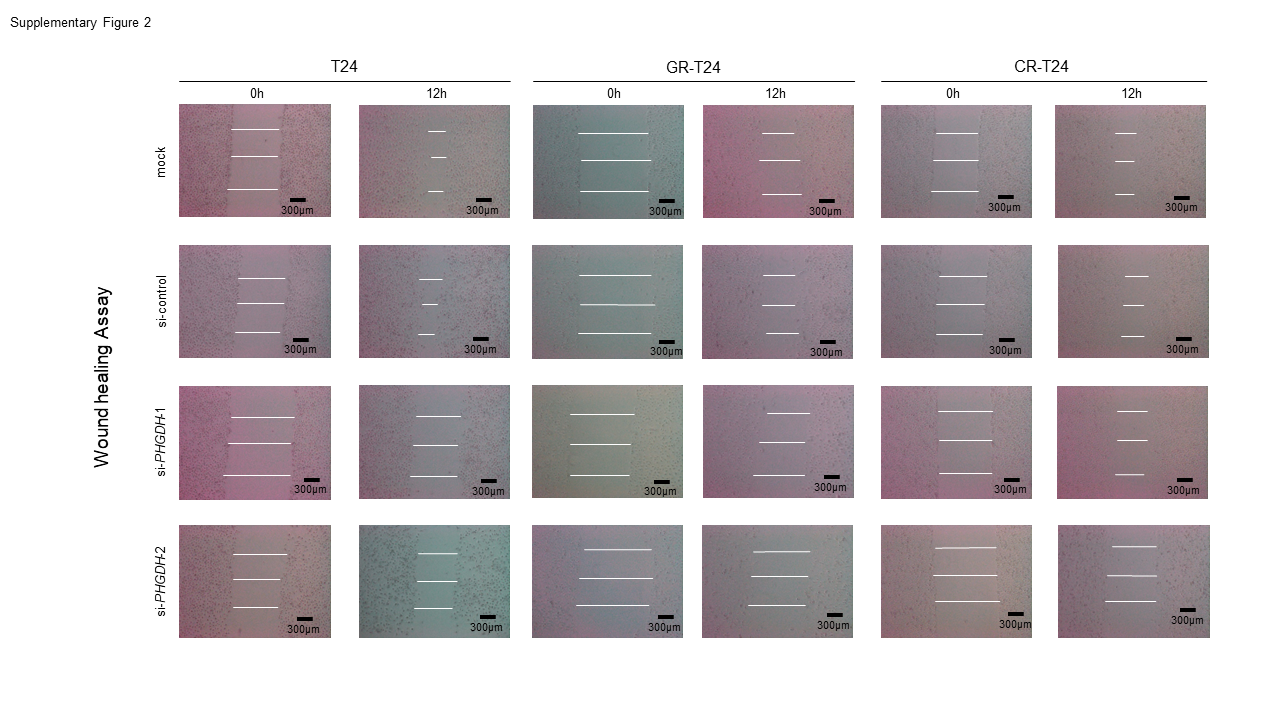

Supplement: Supplementary file 1 — Fig. S1. Pathway analysis of changes in glucose metabolism in parental and resistant bladder cancer cells. Fig. S2. Image of migration assay in parental and gemcitabine‐/cisplatin‐resistant T24 cells after downregulation of PHGDH. Fig. S3. Image of migration assay in parental and gemcitabine‐/cisplatin‐resistant J82 cells after downregulation of PHGDH. Fig. S4. Image of cell invasion assay in drug‐resistant bladder cancer cells after downregulation of PHGDH. Fig. S5. Malignancy and T stage according to PHGDH expression using TCGA data. Fig. S6. Migration and invasion assay in parental and gemcitabine‐/cisplatin‐resistant cells with combination NCT503 and erdafitinib therapy. Fig. S7. Image of migration assay in parental and gemcitabine‐/cisplatin‐resistant T24 cells treated with combination NCT503 and erdafitinib therapy. Fig. S8. Image of migration assay in parental and gemcitabine‐/cisplatin‐resistant J82 cells treated with combination NCT503 and erdafitinib therapy. Fig. S9. Image of invasion assay of parental and resistant cell lines after NCT503 plus erdafitinib combination treatment. Fig. S10. Apoptosis assay of parental and resistant cell lines after NCT503 plus erdafitinib combination treatment. Fig. S11. Western blotting of BAX, p‐Erk, and p‐AKT after NCT503 and erdafitinib therapy. Fig. S12. Body weight changes in mice treated with combination NCT503 and erdafitinib. Fig. S13. Vehicle and NCT503 therapy in a cisplatin‐resistant T24 xenograft mouse model. [file MOL2-18-2196-s001.zip › mol213684-sup-0002-FigS2.TIF]

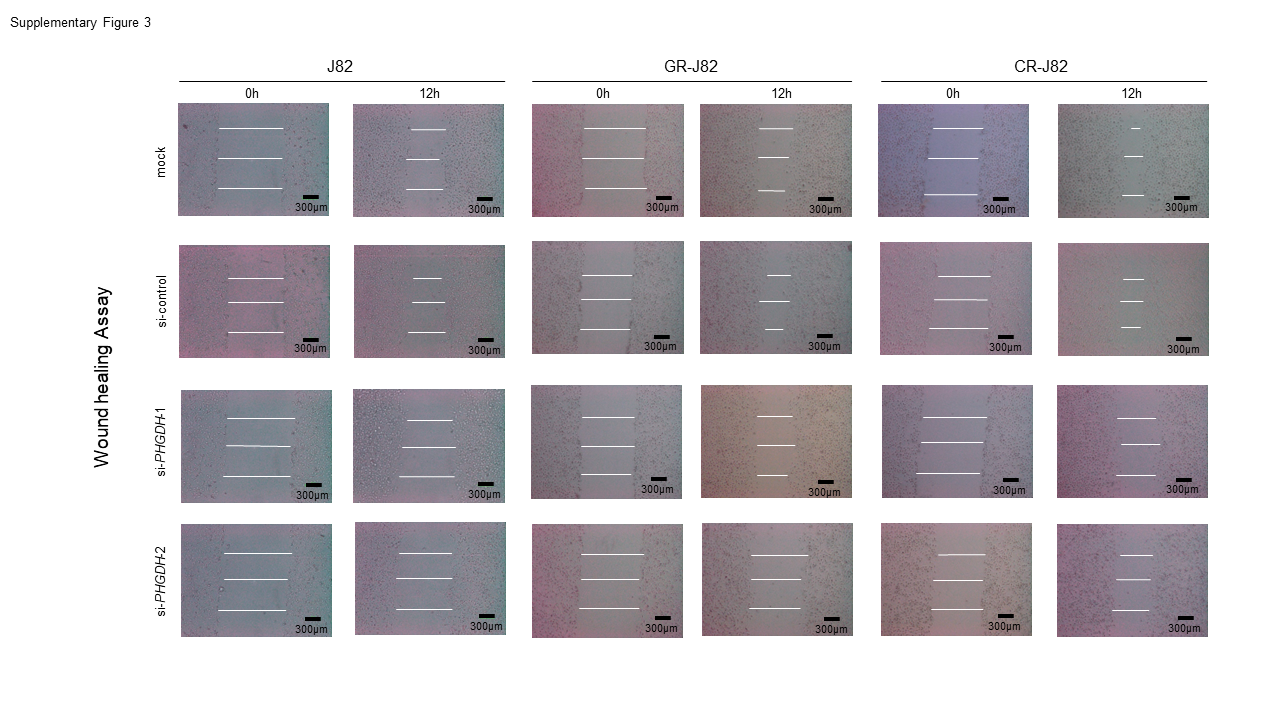

Supplement: Supplementary file 1 — Fig. S1. Pathway analysis of changes in glucose metabolism in parental and resistant bladder cancer cells. Fig. S2. Image of migration assay in parental and gemcitabine‐/cisplatin‐resistant T24 cells after downregulation of PHGDH. Fig. S3. Image of migration assay in parental and gemcitabine‐/cisplatin‐resistant J82 cells after downregulation of PHGDH. Fig. S4. Image of cell invasion assay in drug‐resistant bladder cancer cells after downregulation of PHGDH. Fig. S5. Malignancy and T stage according to PHGDH expression using TCGA data. Fig. S6. Migration and invasion assay in parental and gemcitabine‐/cisplatin‐resistant cells with combination NCT503 and erdafitinib therapy. Fig. S7. Image of migration assay in parental and gemcitabine‐/cisplatin‐resistant T24 cells treated with combination NCT503 and erdafitinib therapy. Fig. S8. Image of migration assay in parental and gemcitabine‐/cisplatin‐resistant J82 cells treated with combination NCT503 and erdafitinib therapy. Fig. S9. Image of invasion assay of parental and resistant cell lines after NCT503 plus erdafitinib combination treatment. Fig. S10. Apoptosis assay of parental and resistant cell lines after NCT503 plus erdafitinib combination treatment. Fig. S11. Western blotting of BAX, p‐Erk, and p‐AKT after NCT503 and erdafitinib therapy. Fig. S12. Body weight changes in mice treated with combination NCT503 and erdafitinib. Fig. S13. Vehicle and NCT503 therapy in a cisplatin‐resistant T24 xenograft mouse model. [file MOL2-18-2196-s001.zip › mol213684-sup-0003-FigS3.TIF]

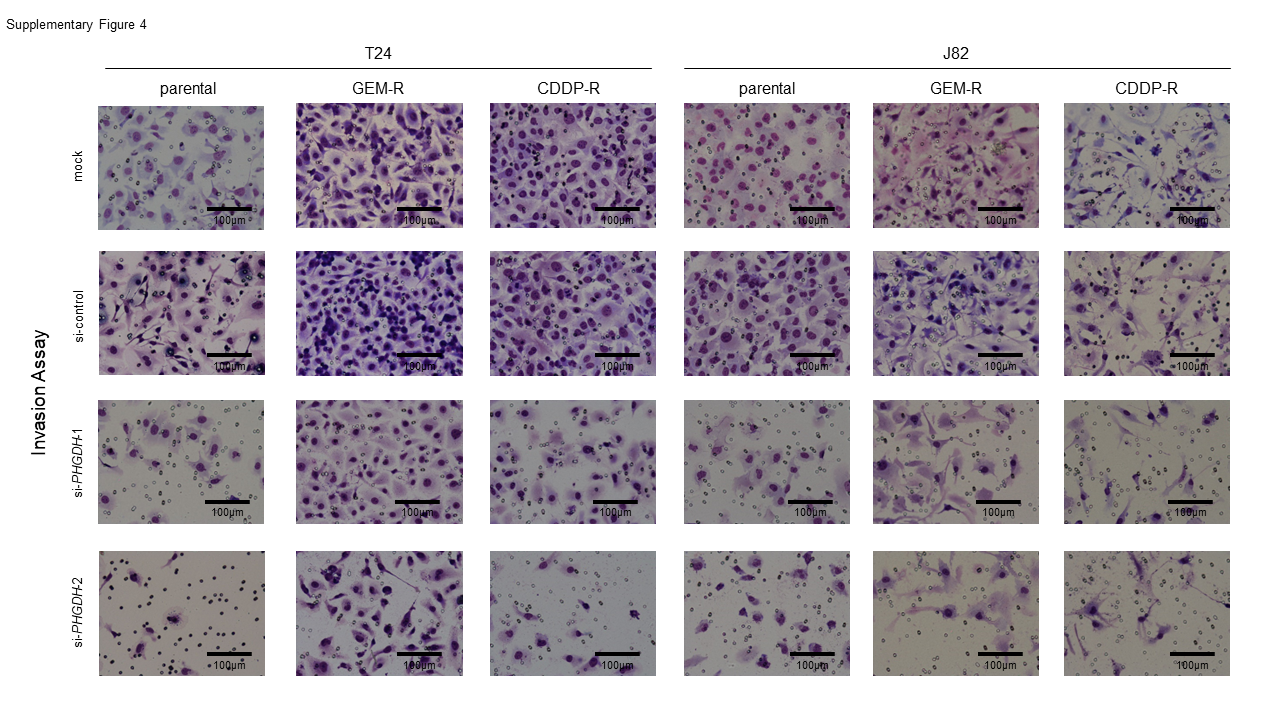

Supplement: Supplementary file 1 — Fig. S1. Pathway analysis of changes in glucose metabolism in parental and resistant bladder cancer cells. Fig. S2. Image of migration assay in parental and gemcitabine‐/cisplatin‐resistant T24 cells after downregulation of PHGDH. Fig. S3. Image of migration assay in parental and gemcitabine‐/cisplatin‐resistant J82 cells after downregulation of PHGDH. Fig. S4. Image of cell invasion assay in drug‐resistant bladder cancer cells after downregulation of PHGDH. Fig. S5. Malignancy and T stage according to PHGDH expression using TCGA data. Fig. S6. Migration and invasion assay in parental and gemcitabine‐/cisplatin‐resistant cells with combination NCT503 and erdafitinib therapy. Fig. S7. Image of migration assay in parental and gemcitabine‐/cisplatin‐resistant T24 cells treated with combination NCT503 and erdafitinib therapy. Fig. S8. Image of migration assay in parental and gemcitabine‐/cisplatin‐resistant J82 cells treated with combination NCT503 and erdafitinib therapy. Fig. S9. Image of invasion assay of parental and resistant cell lines after NCT503 plus erdafitinib combination treatment. Fig. S10. Apoptosis assay of parental and resistant cell lines after NCT503 plus erdafitinib combination treatment. Fig. S11. Western blotting of BAX, p‐Erk, and p‐AKT after NCT503 and erdafitinib therapy. Fig. S12. Body weight changes in mice treated with combination NCT503 and erdafitinib. Fig. S13. Vehicle and NCT503 therapy in a cisplatin‐resistant T24 xenograft mouse model. [file MOL2-18-2196-s001.zip › mol213684-sup-0004-FigS4.TIF]

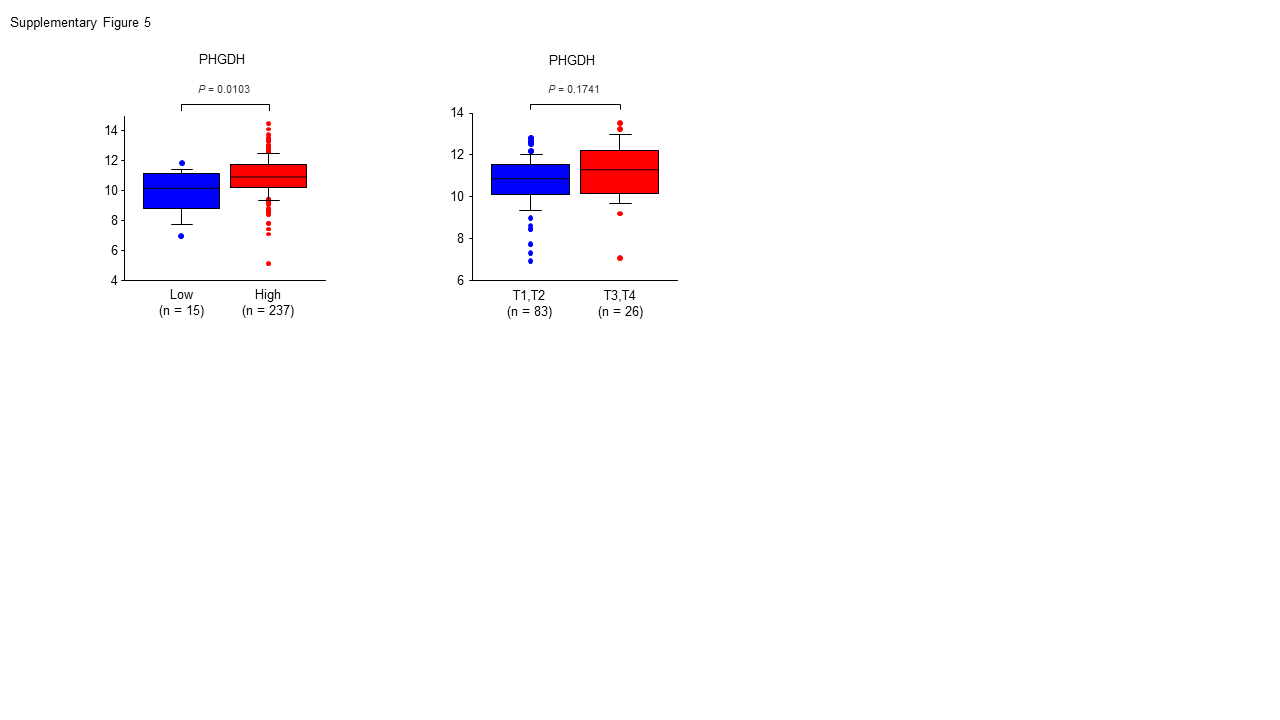

Supplement: Supplementary file 1 — Fig. S1. Pathway analysis of changes in glucose metabolism in parental and resistant bladder cancer cells. Fig. S2. Image of migration assay in parental and gemcitabine‐/cisplatin‐resistant T24 cells after downregulation of PHGDH. Fig. S3. Image of migration assay in parental and gemcitabine‐/cisplatin‐resistant J82 cells after downregulation of PHGDH. Fig. S4. Image of cell invasion assay in drug‐resistant bladder cancer cells after downregulation of PHGDH. Fig. S5. Malignancy and T stage according to PHGDH expression using TCGA data. Fig. S6. Migration and invasion assay in parental and gemcitabine‐/cisplatin‐resistant cells with combination NCT503 and erdafitinib therapy. Fig. S7. Image of migration assay in parental and gemcitabine‐/cisplatin‐resistant T24 cells treated with combination NCT503 and erdafitinib therapy. Fig. S8. Image of migration assay in parental and gemcitabine‐/cisplatin‐resistant J82 cells treated with combination NCT503 and erdafitinib therapy. Fig. S9. Image of invasion assay of parental and resistant cell lines after NCT503 plus erdafitinib combination treatment. Fig. S10. Apoptosis assay of parental and resistant cell lines after NCT503 plus erdafitinib combination treatment. Fig. S11. Western blotting of BAX, p‐Erk, and p‐AKT after NCT503 and erdafitinib therapy. Fig. S12. Body weight changes in mice treated with combination NCT503 and erdafitinib. Fig. S13. Vehicle and NCT503 therapy in a cisplatin‐resistant T24 xenograft mouse model. [file MOL2-18-2196-s001.zip › mol213684-sup-0005-FigS5.TIF]

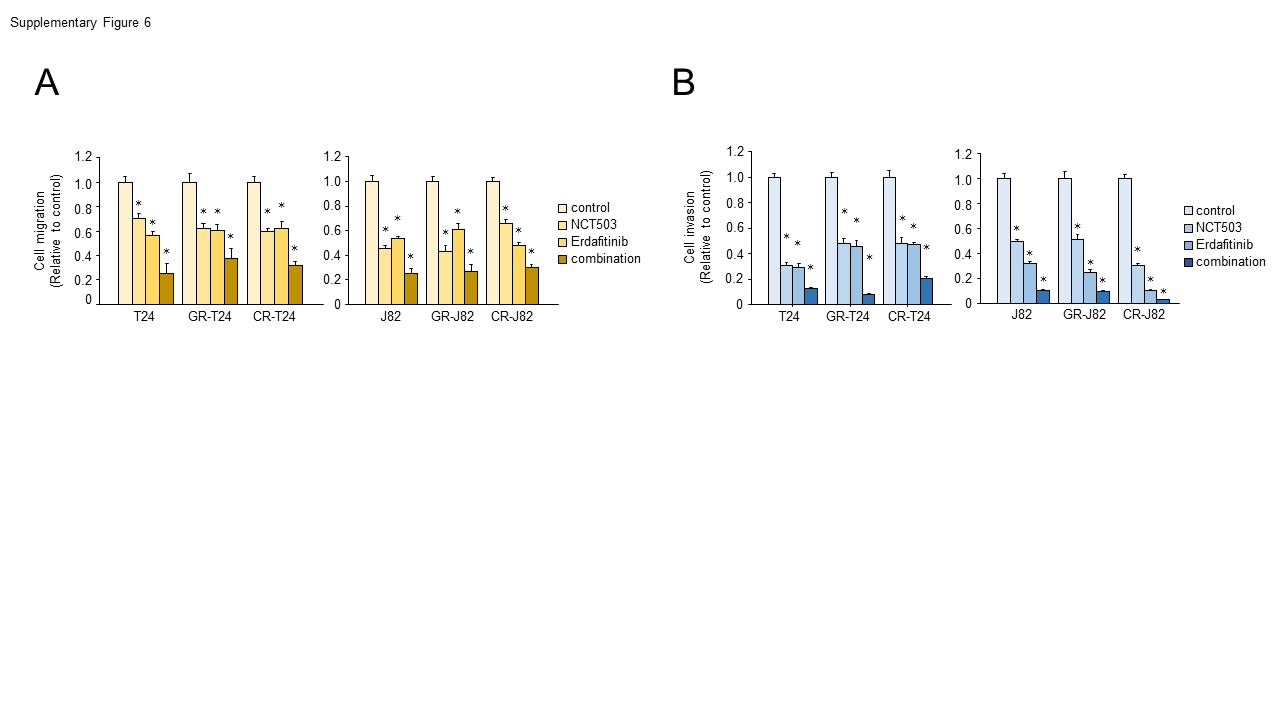

Supplement: Supplementary file 1 — Fig. S1. Pathway analysis of changes in glucose metabolism in parental and resistant bladder cancer cells. Fig. S2. Image of migration assay in parental and gemcitabine‐/cisplatin‐resistant T24 cells after downregulation of PHGDH. Fig. S3. Image of migration assay in parental and gemcitabine‐/cisplatin‐resistant J82 cells after downregulation of PHGDH. Fig. S4. Image of cell invasion assay in drug‐resistant bladder cancer cells after downregulation of PHGDH. Fig. S5. Malignancy and T stage according to PHGDH expression using TCGA data. Fig. S6. Migration and invasion assay in parental and gemcitabine‐/cisplatin‐resistant cells with combination NCT503 and erdafitinib therapy. Fig. S7. Image of migration assay in parental and gemcitabine‐/cisplatin‐resistant T24 cells treated with combination NCT503 and erdafitinib therapy. Fig. S8. Image of migration assay in parental and gemcitabine‐/cisplatin‐resistant J82 cells treated with combination NCT503 and erdafitinib therapy. Fig. S9. Image of invasion assay of parental and resistant cell lines after NCT503 plus erdafitinib combination treatment. Fig. S10. Apoptosis assay of parental and resistant cell lines after NCT503 plus erdafitinib combination treatment. Fig. S11. Western blotting of BAX, p‐Erk, and p‐AKT after NCT503 and erdafitinib therapy. Fig. S12. Body weight changes in mice treated with combination NCT503 and erdafitinib. Fig. S13. Vehicle and NCT503 therapy in a cisplatin‐resistant T24 xenograft mouse model. [file MOL2-18-2196-s001.zip › mol213684-sup-0006-FigS6.TIF]

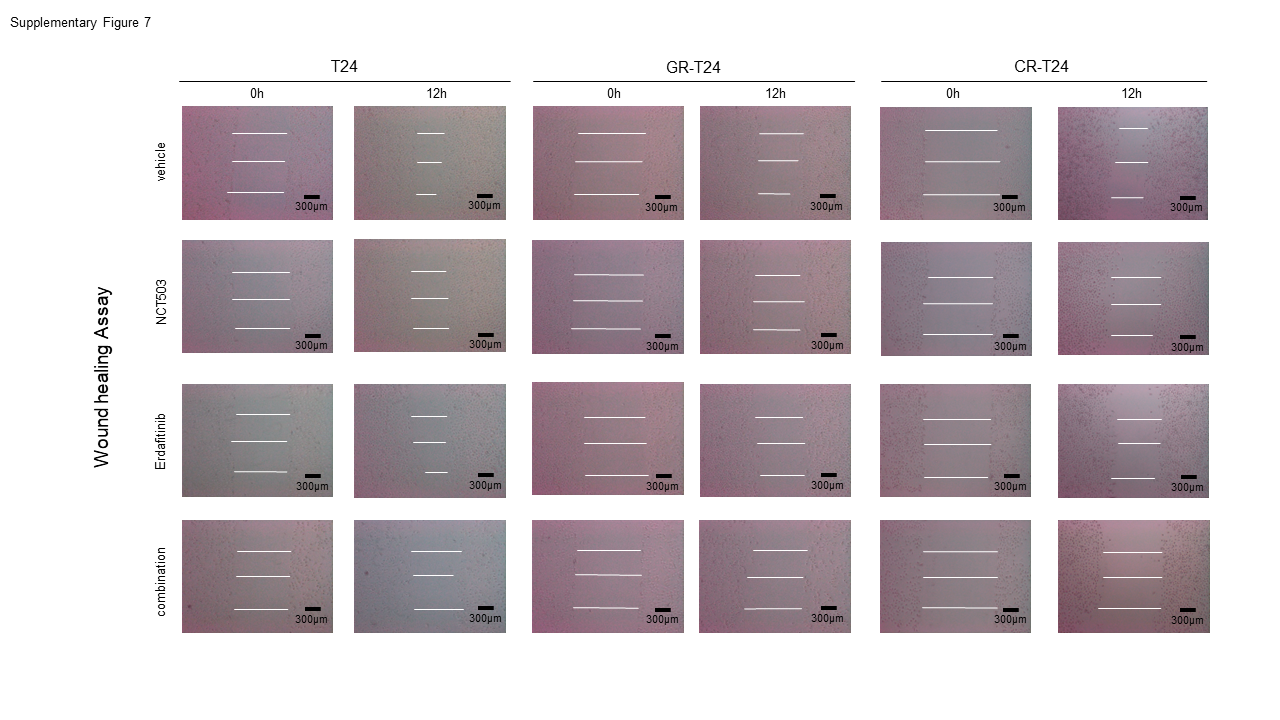

Supplement: Supplementary file 1 — Fig. S1. Pathway analysis of changes in glucose metabolism in parental and resistant bladder cancer cells. Fig. S2. Image of migration assay in parental and gemcitabine‐/cisplatin‐resistant T24 cells after downregulation of PHGDH. Fig. S3. Image of migration assay in parental and gemcitabine‐/cisplatin‐resistant J82 cells after downregulation of PHGDH. Fig. S4. Image of cell invasion assay in drug‐resistant bladder cancer cells after downregulation of PHGDH. Fig. S5. Malignancy and T stage according to PHGDH expression using TCGA data. Fig. S6. Migration and invasion assay in parental and gemcitabine‐/cisplatin‐resistant cells with combination NCT503 and erdafitinib therapy. Fig. S7. Image of migration assay in parental and gemcitabine‐/cisplatin‐resistant T24 cells treated with combination NCT503 and erdafitinib therapy. Fig. S8. Image of migration assay in parental and gemcitabine‐/cisplatin‐resistant J82 cells treated with combination NCT503 and erdafitinib therapy. Fig. S9. Image of invasion assay of parental and resistant cell lines after NCT503 plus erdafitinib combination treatment. Fig. S10. Apoptosis assay of parental and resistant cell lines after NCT503 plus erdafitinib combination treatment. Fig. S11. Western blotting of BAX, p‐Erk, and p‐AKT after NCT503 and erdafitinib therapy. Fig. S12. Body weight changes in mice treated with combination NCT503 and erdafitinib. Fig. S13. Vehicle and NCT503 therapy in a cisplatin‐resistant T24 xenograft mouse model. [file MOL2-18-2196-s001.zip › mol213684-sup-0007-FigS7.TIF]

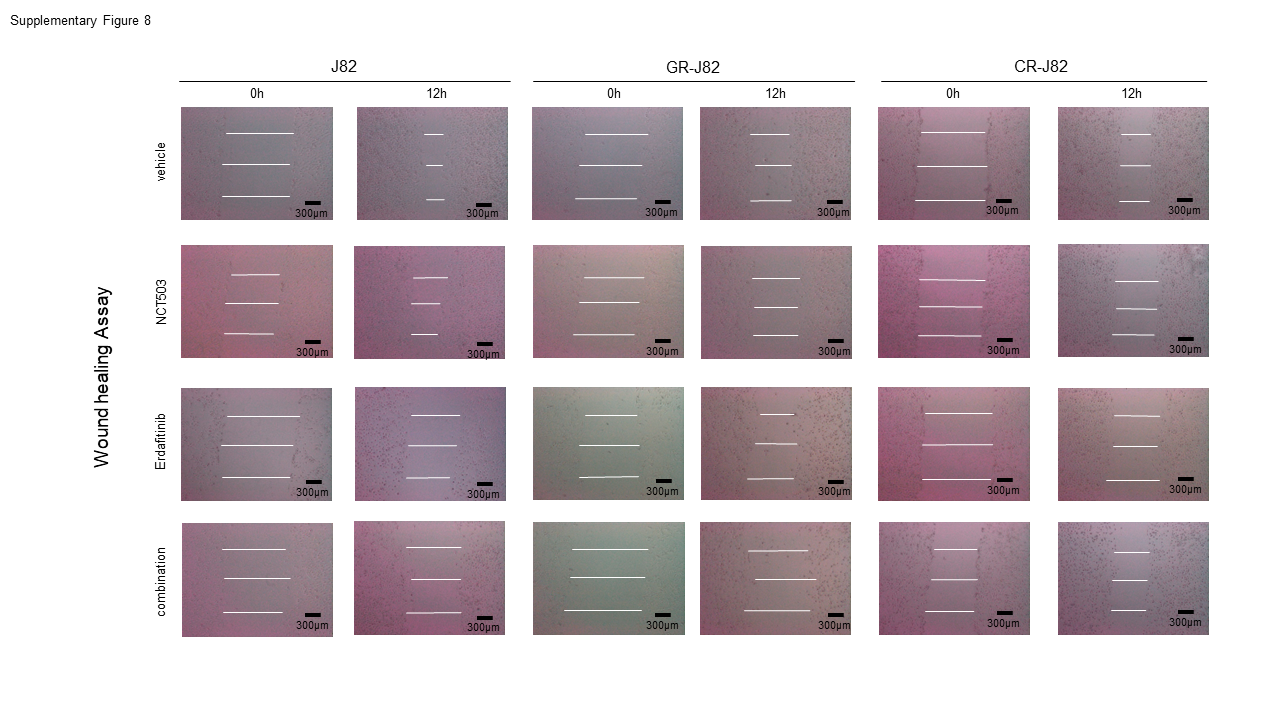

Supplement: Supplementary file 1 — Fig. S1. Pathway analysis of changes in glucose metabolism in parental and resistant bladder cancer cells. Fig. S2. Image of migration assay in parental and gemcitabine‐/cisplatin‐resistant T24 cells after downregulation of PHGDH. Fig. S3. Image of migration assay in parental and gemcitabine‐/cisplatin‐resistant J82 cells after downregulation of PHGDH. Fig. S4. Image of cell invasion assay in drug‐resistant bladder cancer cells after downregulation of PHGDH. Fig. S5. Malignancy and T stage according to PHGDH expression using TCGA data. Fig. S6. Migration and invasion assay in parental and gemcitabine‐/cisplatin‐resistant cells with combination NCT503 and erdafitinib therapy. Fig. S7. Image of migration assay in parental and gemcitabine‐/cisplatin‐resistant T24 cells treated with combination NCT503 and erdafitinib therapy. Fig. S8. Image of migration assay in parental and gemcitabine‐/cisplatin‐resistant J82 cells treated with combination NCT503 and erdafitinib therapy. Fig. S9. Image of invasion assay of parental and resistant cell lines after NCT503 plus erdafitinib combination treatment. Fig. S10. Apoptosis assay of parental and resistant cell lines after NCT503 plus erdafitinib combination treatment. Fig. S11. Western blotting of BAX, p‐Erk, and p‐AKT after NCT503 and erdafitinib therapy. Fig. S12. Body weight changes in mice treated with combination NCT503 and erdafitinib. Fig. S13. Vehicle and NCT503 therapy in a cisplatin‐resistant T24 xenograft mouse model. [file MOL2-18-2196-s001.zip › mol213684-sup-0008-FigS8.TIF]

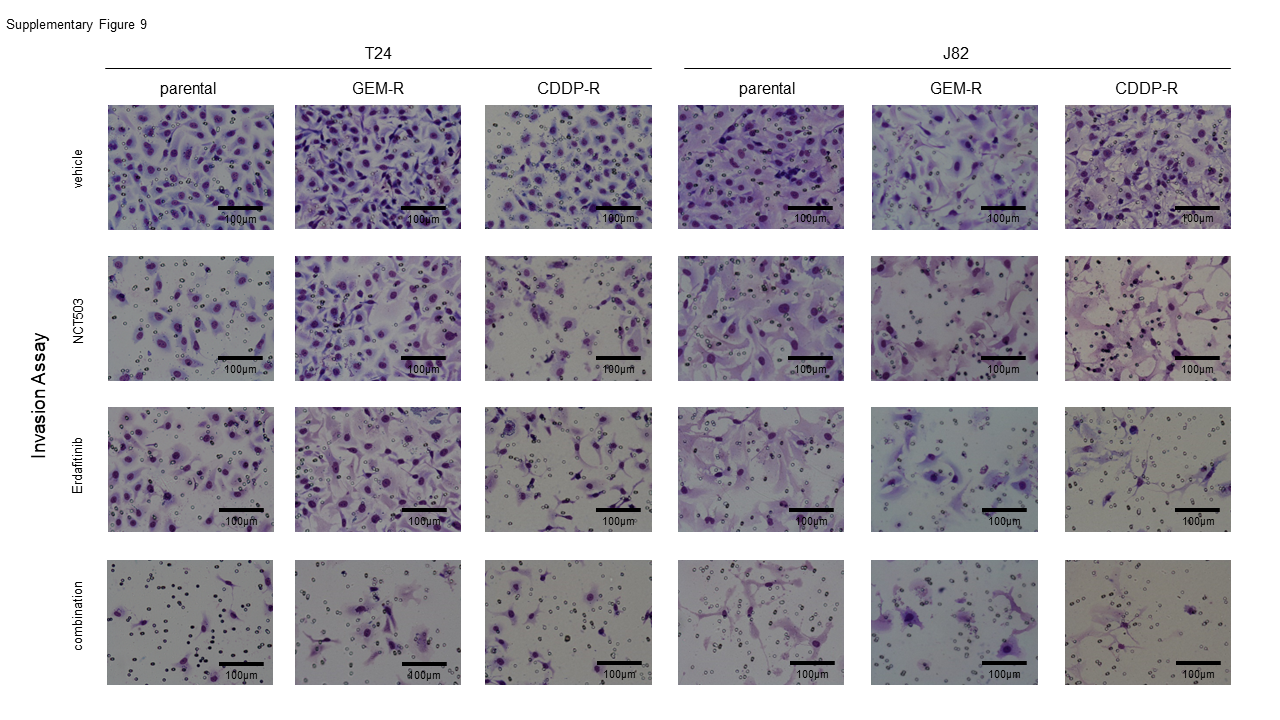

Supplement: Supplementary file 1 — Fig. S1. Pathway analysis of changes in glucose metabolism in parental and resistant bladder cancer cells. Fig. S2. Image of migration assay in parental and gemcitabine‐/cisplatin‐resistant T24 cells after downregulation of PHGDH. Fig. S3. Image of migration assay in parental and gemcitabine‐/cisplatin‐resistant J82 cells after downregulation of PHGDH. Fig. S4. Image of cell invasion assay in drug‐resistant bladder cancer cells after downregulation of PHGDH. Fig. S5. Malignancy and T stage according to PHGDH expression using TCGA data. Fig. S6. Migration and invasion assay in parental and gemcitabine‐/cisplatin‐resistant cells with combination NCT503 and erdafitinib therapy. Fig. S7. Image of migration assay in parental and gemcitabine‐/cisplatin‐resistant T24 cells treated with combination NCT503 and erdafitinib therapy. Fig. S8. Image of migration assay in parental and gemcitabine‐/cisplatin‐resistant J82 cells treated with combination NCT503 and erdafitinib therapy. Fig. S9. Image of invasion assay of parental and resistant cell lines after NCT503 plus erdafitinib combination treatment. Fig. S10. Apoptosis assay of parental and resistant cell lines after NCT503 plus erdafitinib combination treatment. Fig. S11. Western blotting of BAX, p‐Erk, and p‐AKT after NCT503 and erdafitinib therapy. Fig. S12. Body weight changes in mice treated with combination NCT503 and erdafitinib. Fig. S13. Vehicle and NCT503 therapy in a cisplatin‐resistant T24 xenograft mouse model. [file MOL2-18-2196-s001.zip › mol213684-sup-0009-FigS9.TIF]

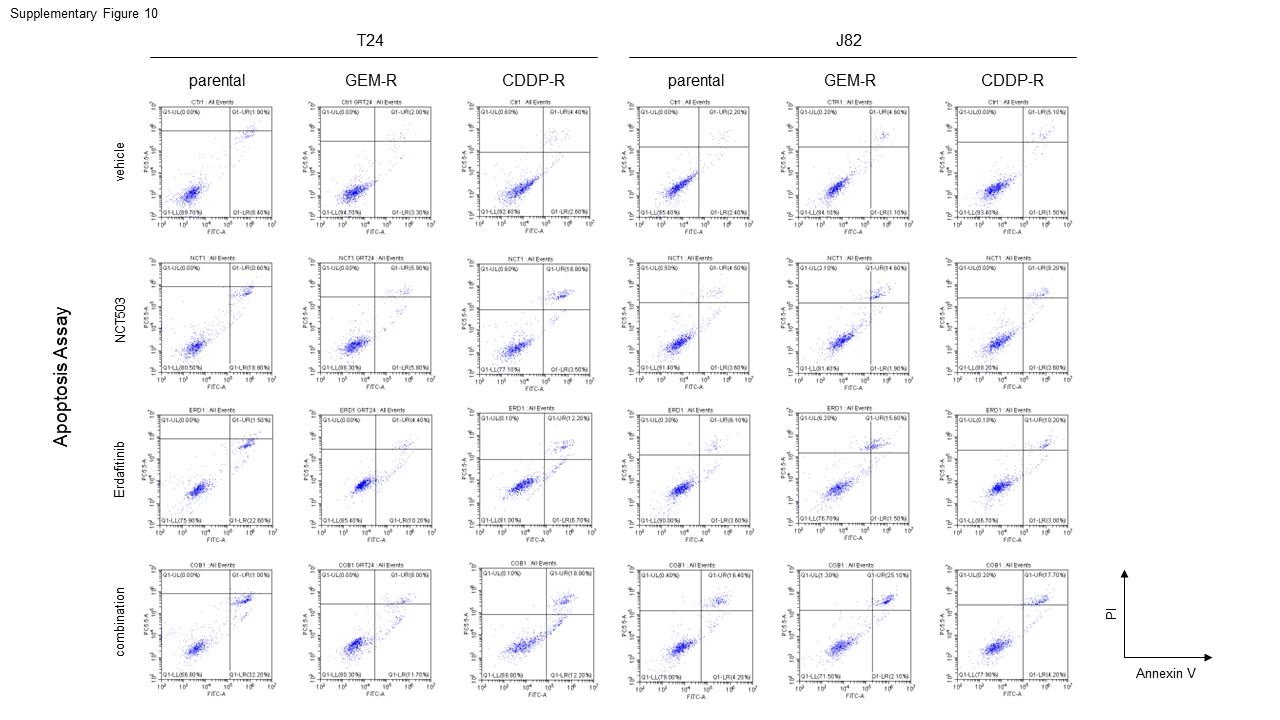

Supplement: Supplementary file 1 — Fig. S1. Pathway analysis of changes in glucose metabolism in parental and resistant bladder cancer cells. Fig. S2. Image of migration assay in parental and gemcitabine‐/cisplatin‐resistant T24 cells after downregulation of PHGDH. Fig. S3. Image of migration assay in parental and gemcitabine‐/cisplatin‐resistant J82 cells after downregulation of PHGDH. Fig. S4. Image of cell invasion assay in drug‐resistant bladder cancer cells after downregulation of PHGDH. Fig. S5. Malignancy and T stage according to PHGDH expression using TCGA data. Fig. S6. Migration and invasion assay in parental and gemcitabine‐/cisplatin‐resistant cells with combination NCT503 and erdafitinib therapy. Fig. S7. Image of migration assay in parental and gemcitabine‐/cisplatin‐resistant T24 cells treated with combination NCT503 and erdafitinib therapy. Fig. S8. Image of migration assay in parental and gemcitabine‐/cisplatin‐resistant J82 cells treated with combination NCT503 and erdafitinib therapy. Fig. S9. Image of invasion assay of parental and resistant cell lines after NCT503 plus erdafitinib combination treatment. Fig. S10. Apoptosis assay of parental and resistant cell lines after NCT503 plus erdafitinib combination treatment. Fig. S11. Western blotting of BAX, p‐Erk, and p‐AKT after NCT503 and erdafitinib therapy. Fig. S12. Body weight changes in mice treated with combination NCT503 and erdafitinib. Fig. S13. Vehicle and NCT503 therapy in a cisplatin‐resistant T24 xenograft mouse model. [file MOL2-18-2196-s001.zip › mol213684-sup-0010-FigS10.TIF]

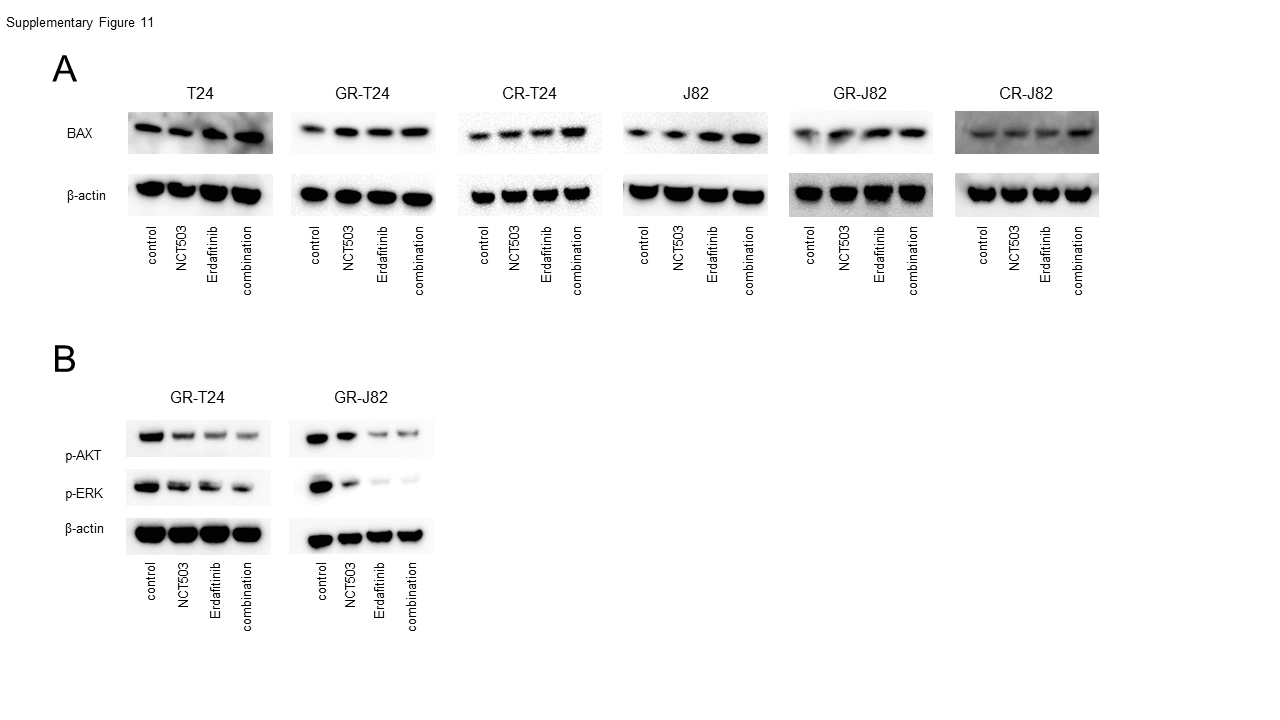

Supplement: Supplementary file 1 — Fig. S1. Pathway analysis of changes in glucose metabolism in parental and resistant bladder cancer cells. Fig. S2. Image of migration assay in parental and gemcitabine‐/cisplatin‐resistant T24 cells after downregulation of PHGDH. Fig. S3. Image of migration assay in parental and gemcitabine‐/cisplatin‐resistant J82 cells after downregulation of PHGDH. Fig. S4. Image of cell invasion assay in drug‐resistant bladder cancer cells after downregulation of PHGDH. Fig. S5. Malignancy and T stage according to PHGDH expression using TCGA data. Fig. S6. Migration and invasion assay in parental and gemcitabine‐/cisplatin‐resistant cells with combination NCT503 and erdafitinib therapy. Fig. S7. Image of migration assay in parental and gemcitabine‐/cisplatin‐resistant T24 cells treated with combination NCT503 and erdafitinib therapy. Fig. S8. Image of migration assay in parental and gemcitabine‐/cisplatin‐resistant J82 cells treated with combination NCT503 and erdafitinib therapy. Fig. S9. Image of invasion assay of parental and resistant cell lines after NCT503 plus erdafitinib combination treatment. Fig. S10. Apoptosis assay of parental and resistant cell lines after NCT503 plus erdafitinib combination treatment. Fig. S11. Western blotting of BAX, p‐Erk, and p‐AKT after NCT503 and erdafitinib therapy. Fig. S12. Body weight changes in mice treated with combination NCT503 and erdafitinib. Fig. S13. Vehicle and NCT503 therapy in a cisplatin‐resistant T24 xenograft mouse model. [file MOL2-18-2196-s001.zip › mol213684-sup-0011-FigS11.TIF]

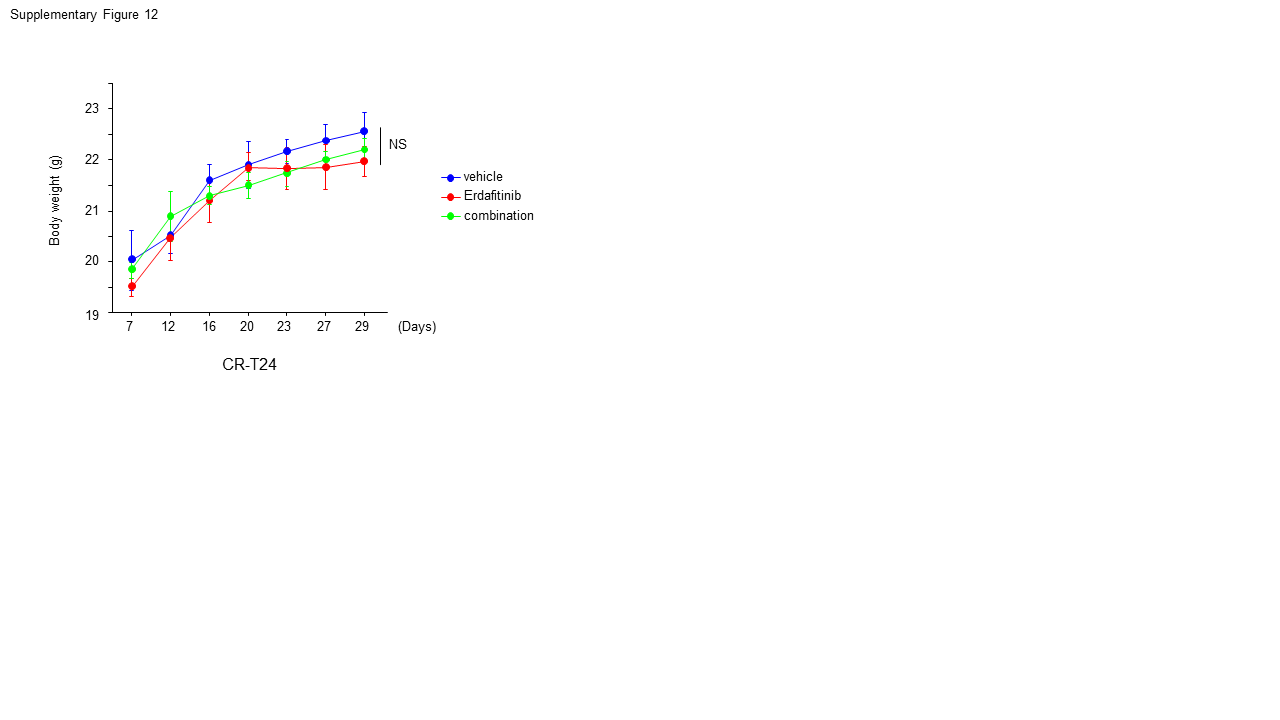

Supplement: Supplementary file 1 — Fig. S1. Pathway analysis of changes in glucose metabolism in parental and resistant bladder cancer cells. Fig. S2. Image of migration assay in parental and gemcitabine‐/cisplatin‐resistant T24 cells after downregulation of PHGDH. Fig. S3. Image of migration assay in parental and gemcitabine‐/cisplatin‐resistant J82 cells after downregulation of PHGDH. Fig. S4. Image of cell invasion assay in drug‐resistant bladder cancer cells after downregulation of PHGDH. Fig. S5. Malignancy and T stage according to PHGDH expression using TCGA data. Fig. S6. Migration and invasion assay in parental and gemcitabine‐/cisplatin‐resistant cells with combination NCT503 and erdafitinib therapy. Fig. S7. Image of migration assay in parental and gemcitabine‐/cisplatin‐resistant T24 cells treated with combination NCT503 and erdafitinib therapy. Fig. S8. Image of migration assay in parental and gemcitabine‐/cisplatin‐resistant J82 cells treated with combination NCT503 and erdafitinib therapy. Fig. S9. Image of invasion assay of parental and resistant cell lines after NCT503 plus erdafitinib combination treatment. Fig. S10. Apoptosis assay of parental and resistant cell lines after NCT503 plus erdafitinib combination treatment. Fig. S11. Western blotting of BAX, p‐Erk, and p‐AKT after NCT503 and erdafitinib therapy. Fig. S12. Body weight changes in mice treated with combination NCT503 and erdafitinib. Fig. S13. Vehicle and NCT503 therapy in a cisplatin‐resistant T24 xenograft mouse model. [file MOL2-18-2196-s001.zip › mol213684-sup-0012-FigS12.TIF]

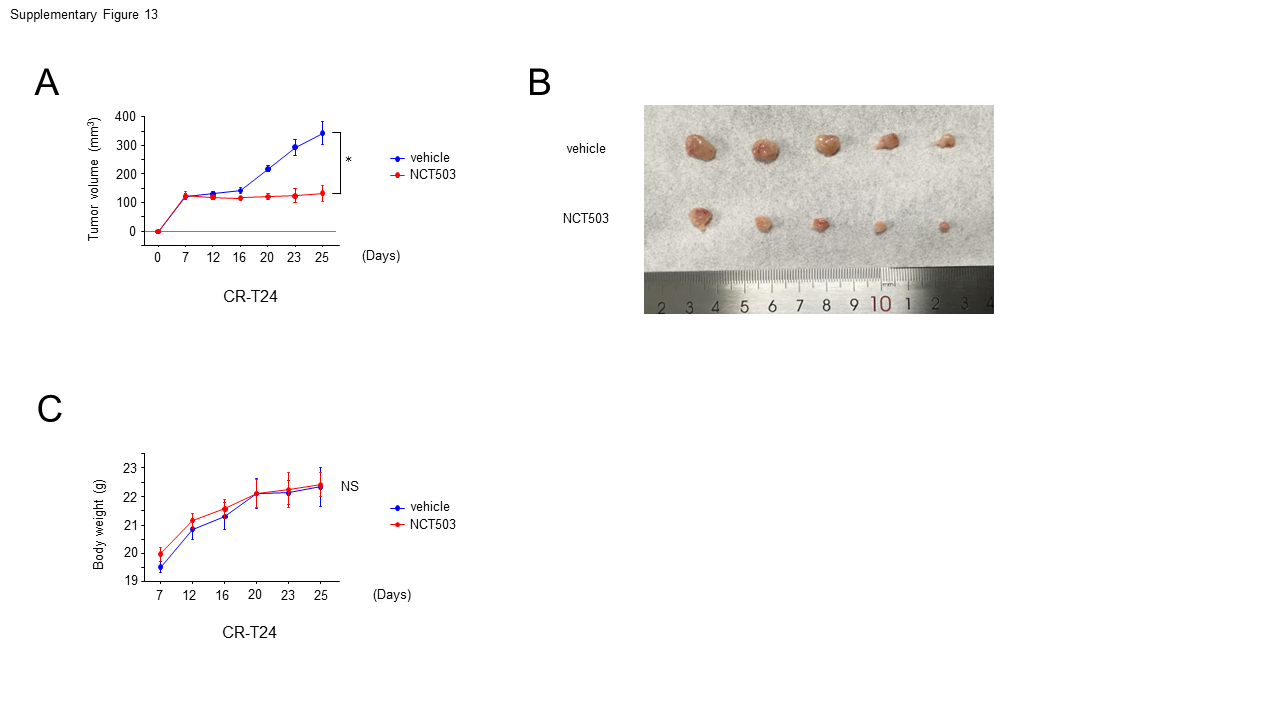

Supplement: Supplementary file 1 — Fig. S1. Pathway analysis of changes in glucose metabolism in parental and resistant bladder cancer cells. Fig. S2. Image of migration assay in parental and gemcitabine‐/cisplatin‐resistant T24 cells after downregulation of PHGDH. Fig. S3. Image of migration assay in parental and gemcitabine‐/cisplatin‐resistant J82 cells after downregulation of PHGDH. Fig. S4. Image of cell invasion assay in drug‐resistant bladder cancer cells after downregulation of PHGDH. Fig. S5. Malignancy and T stage according to PHGDH expression using TCGA data. Fig. S6. Migration and invasion assay in parental and gemcitabine‐/cisplatin‐resistant cells with combination NCT503 and erdafitinib therapy. Fig. S7. Image of migration assay in parental and gemcitabine‐/cisplatin‐resistant T24 cells treated with combination NCT503 and erdafitinib therapy. Fig. S8. Image of migration assay in parental and gemcitabine‐/cisplatin‐resistant J82 cells treated with combination NCT503 and erdafitinib therapy. Fig. S9. Image of invasion assay of parental and resistant cell lines after NCT503 plus erdafitinib combination treatment. Fig. S10. Apoptosis assay of parental and resistant cell lines after NCT503 plus erdafitinib combination treatment. Fig. S11. Western blotting of BAX, p‐Erk, and p‐AKT after NCT503 and erdafitinib therapy. Fig. S12. Body weight changes in mice treated with combination NCT503 and erdafitinib. Fig. S13. Vehicle and NCT503 therapy in a cisplatin‐resistant T24 xenograft mouse model. [file MOL2-18-2196-s001.zip › mol213684-sup-0013-FigS13.TIF]
